# Genetic variation associated with thyroid autoimmunity shapes the systemic immune response to PD-1 checkpoint blockade

**DOI:** 10.1038/s41467-021-23661-4

**Published:** 2021-06-07

**Authors:** Zia Khan, Christian Hammer, Jonathan Carroll, Flavia Di Nucci, Sergio Ley Acosta, Vidya Maiya, Tushar Bhangale, Julie Hunkapiller, Ira Mellman, Matthew L. Albert, Mark I. McCarthy, G. Scott Chandler

**Affiliations:** 1 Genentech, South San Francisco, CA USA; 2 F. Hoffmann-La Roche, Basel, Switzerland; 3 Present Address: insitro, South San Francisco, CA USA

**Keywords:** Cancer genetics, Genetics, Immunotherapy, Risk factors

## Abstract

Activation of systemic immune responses using PD-1 checkpoint inhibitors is an essential approach to cancer therapy. Yet, the extent of benefit relative to risk of immune related adverse events (irAE) varies widely among patients. Here, we study endocrine irAE from 7 clinical trials across 6 cancers where atezolizumab (anti-PD-L1) was combined with chemotherapies and compared to standard of care. We show that atezolizumab-induced thyroid dysfunction is associated with longer survival. We construct a polygenic risk score (PRS) for lifetime risk of hypothyroidism using a GWAS from the UK Biobank and apply this PRS to genetic data collected from 2,616 patients of European ancestry from these trials. Patients with high PRS are at increased risk of atezolizumab-induced thyroid dysfunction and lower risk of death in triple negative breast cancer. Our results indicate that genetic variation associated with thyroid autoimmunity interacts with biological pathways driving the systemic immune response to PD-1 blockade.

## Abstract (web summary)

Endocrinopathies, such as thyroid autoimmunity, are common among patients treated with immune checkpoint inhibitors. Here, by using a polygenic risk score (PRS) derived from a hypothyroidism GWAS, the authors show that cancer patients with high PRS are at increased risk of atezolizumab (anti-PD-L1)-induced thyroid dysfunction, a condition associated with systemic response to PD-1 checkpoint blockade and longer overall survival.

## Introduction

PD-1 checkpoint inhibitors have made significant advances in the treatment of cancer. Considerable progress has been made in identifying the immune mechanisms that are responsible for the therapeutic benefit observed. These include the enhancement of T-cell priming and activation at the level of dendritic cells and the re-invigoration of “exhausted” intra-tumoral T-cells^1,2^. Yet, PD-1 checkpoint inhibitors act systemically and patients can develop immune toxicities and rheumatic complications, termed immune-related adverse events (irAE). Activation of the immune system due to diminished activity of the PD-1 checkpoint is hypothesized to contribute to autoimmunity that increases risk for irAE, but the underlying risk factors and mechanisms are poorly understood^3–5^. In cancer patients treated with PD-1 checkpoint inhibitors, there exists considerable inter-individual variation in tumor response and immune toxicity. Understanding an individual and their tumor’s immunological status, or cancer-immune set point, can explain this variation and identify therapeutic approaches to improve not only efficacy, but also safety^6^. This approach requires consideration of factors both intrinsic and extrinsic to a tumor—including genetic variation that affects the immune system.

Classes of dermatological and endocrine system irAE that occur during treatment with PD-1 checkpoint inhibitors as monotherapy have been associated with longer survival in melanoma suggesting these irAE may also reflect immune responses associated with anti-tumor immunity^7,8^. A recent study has confirmed these findings by accounting for sources of survival bias that arise when using adverse event data and by comparison to matched melanoma patients receiving placebo^9^. Yet, the clinical development path of PD-1 checkpoint inhibitors has focused on combination therapies. Recent clinical trials have demonstrated that adding immunotherapy to chemotherapy can improve clinical outcomes across cancers - supporting the notion that immune activation provides benefit beyond inducing cell death and inhibiting angiogenesis in tumors.

Endocrinopathies are common among patients that receive immune checkpoint inhibitors and rarely occur in patients treated with chemotherapies. While most dermatological irAE are reversible, endocrinopathies can be permanent - representing a distinct risk for a cancer patient^10^. Studies have identified physiological risk factors for endocrine irAE, yet the general applicability of these findings to chemotherapy combinations and underlying genes and mechanisms remain unclear^11–13^. Derived from disease-associated genomic loci, PRS have the potential to address these limitations. We recently introduced the use of PRS to determine the extent to which genetic variation shared with autoimmune disease impacts dermatological irAE and survival in a single atezolizumab monotherapy bladder cancer trial^14^. Here, we show that thyroid irAE are associated with lower risk of death across cancers and chemotherapy combinations with atezolizumab. To gain further insight into these irAE, we use a PRS derived from a hypothyroidism GWAS to demonstrate that genetic variation affecting lifetime risk for autoimmune thyroid disease also impacts risk of thyroid irAE during the shorter duration of atezolizumab treatment. Sparse regression analysis indicates a subset of variants in this PRS drive this association and are in loci near genes involved in autoimmunity and regulation of immune responses, including responses driven by T-follicular-helper (Tfh) cells. The PRS was directly associated with lower risk of death in triple-negative breast cancer (TNBC) patients treated with atezolizumab and nab-paclitaxel suggesting that immune mechanisms that lead to thyroid autoimmunity may improve survival in these patients. Our study emphasizes the importance of genetic variation affecting the immune system during cancer immunotherapy with implications for the use of PRS in clinical decision making and for the prioritization of immunotherapy targets.

## Results

### Thyroid irAE are common and associated with longer survival

**Fig. 1:**
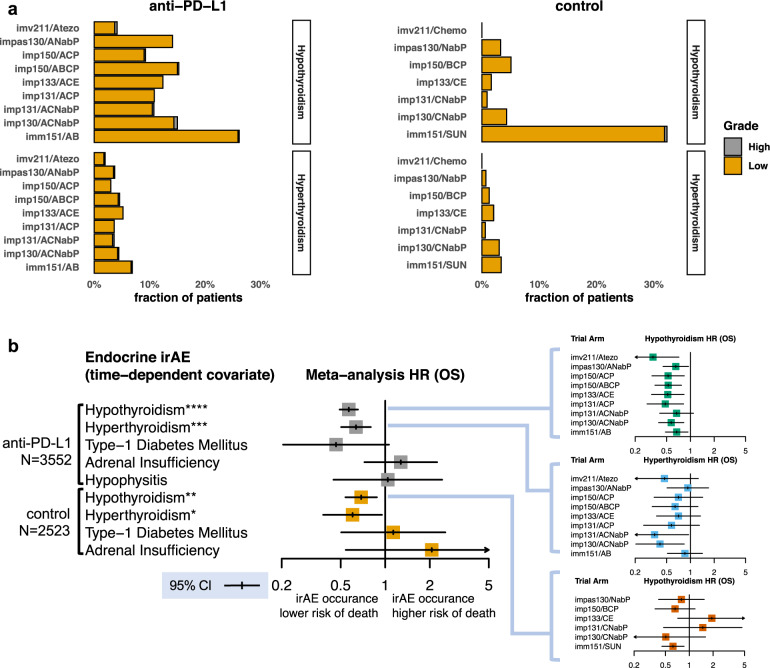
Thyroid irAE are common and associated with longer overall survival during treatment with atezolizumab as monotherapy or in combination. **a** Fraction of patients that developed hypothyroidism and hyperthyroidism irAE across atezolizumab trials and their corresponding control arms. Low designates Common Terminology Criteria for Adverse Events (CTCAE) grading of 1 and 2. High designates CTCAE grade >2. **b** Left, results of an individual participant data (IPD) meta-analysis showing the 95% confidence intervals (CI) and point estimate for a time-dependent covariate in a Cox model associating occurrence of a given endocrine irAE and overall survival in patients treated with atezolizumab (*N* = 3552) or with standard of care treatments in the control arms (*N* = 2523) across 7 clinical trials from the safety evaluable population. IPD meta-analysis *p*-values for a two-sided Wald test that the logarithm (log) of the random effect estimate of a time-dependent covariate in a Cox model, stratified across trials, is non-zero for hypothyroidism *p* = 5.26 × 10^−15^, hyperthyroidism *p* = 1.02 × 10^−4^, type-1 diabetes *p* = 0.06, adrenal insufficiency *p* = 0.4, and hypophysitis *p* = 0.93 in atezolizumab treated patients. IPD meta-analysis *p*-values for patients in the control arms for hypothyroidism *p* = 0.0039, hyperthyroidism *p* = 0.03, type-1 diabetes *p* = 0.76, and adrenal insufficiency *p* = 0.29 obtained by the same test. Subpanels to the right show the 95% CI around the point estimate of the HR for this time-dependent covariate for hyperthyroidism and hypothyroidism split by each individual trial arm. Trials names are abbreviated as follows: imv211 = IMvigor211; impXXX = IMpowerXXX; imm151 = IMmotion151; impas130 = IMpassion130. Abbreviations for treatment combinations are coded as follows: Atezo = atezolizumab monotherapy; A = atezolizumab; C = carboplatin; P = paclitaxel; NabP = Nab-paclitaxel; B = bevacizumab; SUN = sunitinib; E = etoposide. Meta-analysis: *p < 0.05, **p < 0.01, ***p < 0.001, ****p < 0.0001.

We aggregated endocrine system irAE across seven phase 3 trials testing atezolizumab combinations with chemotherapies and bevacizumab spanning six cancer indications: metastatic urothelial carcinoma (IMvigor211^15^), squamous and non-squamous non-small cell lung cancer (IMpower150^16^, IMpower130^17^, IMpower131^18^), small cell lung cancer (IMpower133^19^), metastatic renal cell carcinoma (IMmotion151^20^), and triple-negative breast cancer (TNBC) (IMpassion130^21,22^) (Supplementary Tables 1–2). A total of 6075 patients were in the safety evaluable population in these trials of which *N* = 3552 received atezolizumab in combination or as monotherapy. Consistent with prior reporting, adrenal insufficiency, type-1 diabetes mellitus (T1D), and hypophysitis were infrequent, occurring in 0–0.17% of atezolizumab treated patients, and the probability of occurrence of these events was significantly less than 2% after 1.5 years from the start of treatment (Supplementary Figs. 1–2). In contrast, thyroid irAE, both hypothyroidism and hyperthyroidism, were common (3–26%) and consistent with results reported for other immune checkpoint inhibitors (Fig. 1a)^23^.

To assess the accuracy with which thyroid irAE were identified in these trials, we examined patient thyroid-stimulating hormone (TSH) lab measurements over the course of treatment. The fraction of patients with symptomatic hypothyroidism irAE were a subset of those that experienced abnormal TSH level (>5 mU/L) events, and yet, nearly all symptomatic events were preceded, within 7 days, by an abnormal TSH measurement (see “Methods” and Supplementary Fig. 3). We additionally considered that inflammation of the thyroid can initially appear as hyperthyroidism eventually leading to hypothyroidism as thyroid function is impaired^24^. We assessed whether such a temporal pattern was present in anti-PD-L1 treated patients by using the time at which these events were observed after the start of treatment. Combining all the patients treated with atezolizumab, the plateau in cumulative event probability occurred earlier for hyperthyroidism than for hypothyroidism (Supplementary Fig. 4). Out of 473 atezolizumab treated patients that developed hypothyroidism, we identified 70 where both thyroid dysfunction events were reported. In 85.7% (60/70) of these patients, hyperthyroidism was diagnosed before hypothyroidism. Overall, this analysis indicates that the thyroid irAE data were consistent with expectations of their clinical course.

Treatment with atezolizumab as a monotherapy or in combination was associated with increased risk of hyperthyroidism and hypothyroidism as compared to patients receiving chemotherapies (Fig. 1a). An exception was the sunitinib arm of IMmotion151 which accounted for 74% (144/195) of the hypothyroidism events in the control arms. Sunitinib, a small molecule receptor tyrosine kinase inhibitor, is also known to induce hypothyroidism in treated patients^25^. Because of the use of sunitinib in the control arm, thyroid function was monitored at a higher frequency, possibly accounting for the higher fraction of hypothyroid patients that were identified in both arms of IMmotion151 (see “Methods”). Risk of sunitinib induced hypothyroidism increased with the duration of treatment, a pattern not observed in atezolizumab treated patients as the probability of developing hypothyroidism after 500 days from the start of treatment was significantly higher in sunitinib treated patients than that of the atezolizumab combinations (Supplementary Fig. 5). We estimated that patients were 2.49 (95% CI 1.72–3.60) times more likely to develop hyperthyroidism, and excluding IMmotion151, 3.77 (95% CI 2.39–5.93) times more likely to develop hypothyroidism during treatment with atezolizumab as monotherapy or in combination as compared to the control arms.

Prior studies have identified associations with endocrine irAE in cancer patients and longer overall survival (OS) during monotherapy treatment with a PD-1 checkpoint inhibitor^11–13^. Yet, not all of these studies considered that irAE occur past the point of randomization, introducing the potential for survival bias. Additionally, whether this association holds when PD-1 checkpoint inhibitors are combined with chemotherapies has not been tested. Using a time-dependent covariate in a Cox proportional hazard’s model to address survival bias (see “Methods”), we found that both hypothyroidism (meta-analysis *p* = 5.26 × 10^−15^, HR = 0.57, 95% CI 0.49–0.65) and hyperthyroidism (meta-analysis *p* = 1.02 × 10^−4^, HR = 0.63, 95% CI 0.50–0.79) were associated with longer OS during atezolizumab treatment both as monotherapy and in combination with chemotherapies (Fig. 1b). This was confirmed by landmark analysis as hypothyroidism irAE occurring in the first 5 months (*p* = 0.0024, HR = 0.74, 95% CI 0.56–0.99) were also associated with longer OS. We observed no statistically significant associations with type-1 diabetes mellitus (*p* = 0.067, HR = 0.46, 95% CI 0.20–1.05), adrenal insufficiency (*p* = 0.40, HR = 1.27, 95% CI 0.72–2.24), and hypophysitis (*p* = 0.92, HR = 1.04, 95% CI 0.45–2.42) and OS. As these events were rare (Supplementary Fig. 1), statistical power to detect a general association with endocrinopathies and OS was limited. The 95% confidence interval suggests an association may additionally exist between type-1 diabetes in atezolizumab treated patients and longer OS, but further data is needed to validate this observation (Fig. 1b). Last, confirming prior studies, but also accounting for survival bias, we found a weaker association between OS and hypothyroidism in patients undergoing sunitinib treatment (*p* = 0.0039, HR = 0.62, 95% CI 0.45–0.85)^25^.

### Shared genetic etiology between hypothyroidism and thyroid irAE

Hypothyroidism also occurs in human populations in otherwise healthy individuals—not receiving immune checkpoint inhibitors, bevacizumab, or chemotherapies—over the course of a lifetime due to environmental exposures and genetic factors. Hypothyroidism has a population prevalence estimated to be about 5% in Europeans^24^. Given its prevalence, GWAS have previously been conducted to identify loci associated with lifetime risk. These studies have confirmed that the majority of hypothyroidism in Europeans is caused by autoimmunity as many of the loci are found near genes involved in immune regulation and in GWAS of other autoimmune diseases^26^. One of the largest of these GWAS used data from the UK Biobank^27^. We repeated this GWAS of 25,072 self-reported and ICD code diagnosed hypothyroidism cases and 383,887 controls using SAIGE, a method that better controls type-1 error in case-control imbalanced GWAS (Supplementary Table 3; see “Methods”)^28^. Using LD score regression, we estimated that the heritability captured by this GWAS was 13.1%^29^. We found that this heritability was significantly enriched in cell type specific accessible chromatin as measured by ATAC-seq in CD4^+^ T-cells, CD8^+^ T-cells, and B-cells reflecting the contribution of immune cells involved in autoimmunity (Supplementary Fig. 6). We additionally performed fine mapping and identified 140 independent genome-wide significant signals and computed credible sets which accounted for 99% of the posterior probability of association (PPA) for a given signal (Supplementary Data 1; Supplementary Fig. 7).

**Fig. 2:**
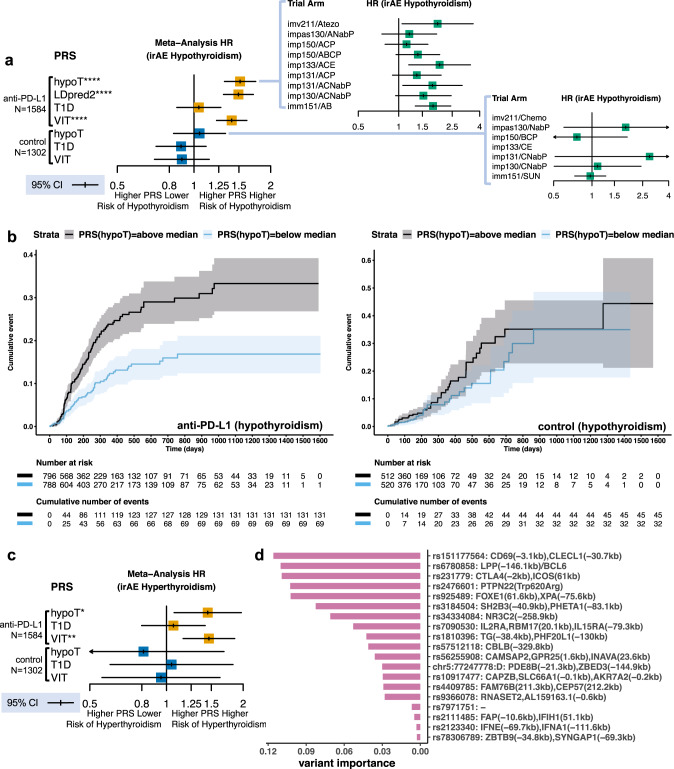
Genetic variation associated with lifetime risk of thyroid autoimmunity contributes to risk of thyroid irAE in atezolizumab treated cancer patients. **a** Random effect point estimate and 95% CI for IPD meta-analysis HR expressed in unit normalized PRS for the occurrence of hypothyroidism irAE estimated in a mixed effect Cox model with genotype eigenvectors as fixed effect covariates using data from (*N* = 1584) atezolizumab and (*N* = 1302) standard of care treated European ancestry cancer patients across 7 clinical trials. PRSs are abbreviated as follows: hypoT = hypothyroidism; LDpred2 = hypothyroidism PRS constructed by beta-shrinkage; T1D = type-1 diabetes; VIT = vitiligo. IPD meta-analysis *p*-values for a two-sided Wald test that the mixed effect Cox model estimated log-HR is non-zero for hypoT *p* = 7.52 × 10^−9^, LDpred2 *p* = 5.49 × 10^−9^, T1D *p* = 0.67, and VIT *p* = 1.10 × 10^−6^ in atezolizumab treated patients and for hypoT *p* = 0.68, T1D *p* = 0.31, and VIT *p* = 0.38 in the control arms stratified by arm. Subpanels show the HR also expressed in unit PRS estimated using a univariable Cox model for each trial arm. **b** Left, cumulative incidence plot comparing risk of hypothyroidism irAE in patients treated with atezolizumab with above and below-median values for the hypothyroidism PRS. The median value was computed across all patients including those in the control arms. Right, cumulative incidence for the same comparison of patients in the control arms. Shaded regions provide the 95% CI. **c** Random effect point estimate and 95% CI for the IPD meta-analysis HR expressed in normalized unit PRS for the time to occurrence of hyperthyroidism irAE estimated using a mixed effect Cox model with genotype eigenvectors as fixed effect covariates in (*N* = 1584) atezolizumab and (*N* = 1302) standard of care treated European ancestry cancer patients across 7 clinical trials. Meta-analysis *p*-values for a two-sided Wald test that the estimated log-HR is non-zero for hypoT *p* = 0.016, T1D *p* = 0.67, and VIT *p* = 0.0012 in atezolizumab treated patients and hypoT *p* = 0.57, T1D *p* = 0.87, and VIT *p* = 0.85 in standard of care treated patients stratified by trial arm. **d** Estimated importance of variants from the hypothyroidism PRS retained in a survival lasso model for time to hypothyroidism irAE in atezolizumab treated patients. The genes whose transcription start sites (TSS) are spanned by the credible set to which the lasso retained variant belongs are provided with no trailing parentheses. The two closest genes in genomic distance to a TSS from credible set ends are indicated by trailing parenthesis containing distance in kilobases (kb). Only genes who have TSS within 500 kb are reported. Dash designates credible sets that span more than 3 TSS. Trials names are abbreviated as follows: imv211 = IMvigor211; impXXX = IMpowerXXX; imm151 = IMmotion151; impas130 = IMpassion130. Abbreviations for treatment combinations are coded as follows: Atezo = atezolizumab monotherapy; A = atezolizumab; C = carboplatin; P = paclitaxel; Nab P = Nab-paclitaxel; B = bevacizumab; SUN = sunitinib; E = etoposide. Meta-analysis: *p < 0.05, **p < 0.01, ***p < 0.001, ****p < 0.0001.

We hypothesized that genetic variation affecting the immune system to alter lifetime hypothyroidism risk also impacts risk of thyroid irAE during the shorter duration of atezolizumab treatment. We tested this hypothesis by constructing a PRS using 140 variants with the highest PPA for each of the genetic credible sets obtained by fine mapping (see “Methods”**;** Supplementary Data 2). We collected and analyzed 30× whole genome sequencing data from 2616 patients, of which 1584 were treated with atezolizumab in combination or as monotherapy, provided informed consent for genetic data collection, were of European ancestry (to match the ancestry of the population used to construct the PRS), and met sample and population quality control filters (see “Methods”; Supplementary Table 2**;** Supplementary Fig. 8). We applied the UK Biobank derived hypothyroidism PRS to time to irAE data from these patients. We found that a higher PRS was associated with increased risk of hypothyroidism irAE in atezolizumab treated cancer patients (meta-analysis, *p* = 7.52 × 10^−9^, HR = 1.52, 95% CI 1.31–1.74 per unit normalized PRS) (Fig. 2a, b).

We tested the robustness of the association between genetic variation and hypothyroidism irAE in atezolizumab treated patients using several approaches. As our 140 variant PRS included only genome-wide significant signals, we constructed a second PRS that consisted of 1,099,649 HapMap3 variants using LDpred2-auto, a method that relies on beta-shrinkage to incorporate signals below genome-wide significance (Supplementary Data 3)^30^. Using the LDpred2-auto PRS, we confirmed that the finding was robust to the underlying method for PRS construction (meta-analysis *p* = 5.49 × 10^−9^, HR = 1.49, 95% CI 1.30–1.71 per unit normalized PRS). We additionally confirmed that the hypothyroidism PRS was associated with a broader event class, time to first abnormal TSH lab measurement (>5 mU/L) (meta-analysis, *p* = 1.31 × 10^−9^, HR = 1.40, 95% CI 1.25–1.56 per unit normalized PRS), in atezolizumab treated patients. Across trials, the association was not correlated with the frequency of thyroid lab testing (sub-panel Fig. 2a). Reflecting their temporal ordering, we found that a higher hypothyroidism PRS was also associated with increased risk of hyperthyroidism irAE (meta-analysis, *p* = 0.016, HR = 1.45, 95% CI 1.07–1.96 per unit normalized PRS, Fig. 2c). Thus, shared genetic factors contribute to risk of atezolizumab-induced hypothyroidism irAE, which occur during cancer treatment, and to risk of hypothyroidism that occurs over a lifetime in European populations.

Patients in the control arms received combinations of chemotherapies, bevacizumab, or sunitinib reflecting standard of care for the cancer indication in which the trial was conducted. In contrast to atezolizumab, which targets the immune system, the drugs in the control arms act to induce death in proliferating cells, inhibit angiogenesis, or both. Combining all of the control arms, we found no association with the PRS and risk of hypothyroidism (meta-analysis, *p* = 0.67; HR = 1.05, 95% CI 0.83–1.11 per unit normalized PRS) indicating that genetic variation affecting the immune system did not alter risk of this event during treatment with these drugs (Fig. 2a, b). The majority of the hypothyroidism events (53/77) in European patients with genetic data from the control arms occurred in the sunitinib arm of IMmotion151. We separately confirmed that the hypothyroidism PRS was not associated with risk of sunitinib induced hypothyroidism (subpanel Fig. 2a, Supplementary Fig. 9). The proportion of patients that developed hypothyroidism during sunitinib treatment was significantly higher than population prevalence, and prior studies have observed high rates of sunitinib induced hypothyroidism in cancer patients^25^. These observations indicate that the absence of an association with PRS in sunitinib induced hypothyroidism can be explained by its differing mechanism of action—receptor tyrosine kinase inhibition—as compared to atezolizumab.

Excluding sunitinib, 24 hypothyroidism events were observed in 843 European chemotherapy treated cancer patients with genetic data in the control arms. Given that the proportion of hypothyroidism events was below the population prevalence and these chemotherapies have not been associated with high rates of hypothyroidism during treatment, these events were likely lifetime cases found due to elevated monitoring and lab testing as per the protocols of the trials. While one might expect the hypothyroidism PRS to be associated with these events, we found no evidence for such an association (Supplementary Fig. 10) and no difference in the PRS values between hypothyroid and non-hypothyroid (*t*-test, *p* = 0.24) chemotherapy treated cancer patients. To determine if this was due to lack of power, we used cross validation to estimate the effect size of the hypothyroidism PRS between lifetime cases and controls in in the UK Biobank (see “Methods”). We found that the power to detect a difference between these 24 cases and 819 controls was limited (power = 0.4 at a significance level of 0.01). Furthermore, assuming a similar proportion of such cases in atezolizumab treated patients, we had little or no power to detect a difference (power < 0.01) at a significance level we observed for the hypothyroidism PRS and irAE association. Taken together, this analysis indicates that a small fraction of lifetime hypothyroidism cases are found in the trials studied, but do not account for the association we observe between the hypothyroidism PRS and atezolizumab-induced hypothyroidism irAE.

Autoimmune disorders, including T1D and vitiligo, co-occur with thyroid dysfunction including hyperthyroidism and hypothyroidism over the course of a lifetime^31^. GWAS have also been conducted for these disorders to identify risk loci shared with hypothyroidism. Therefore, we asked if genetic variation ascertained by these studies was also associated with risk of thyroid irAE during cancer immunotherapy treatment. To test this, we constructed PRS for T1D and vitiligo using a consistent methodology and summary statistics from the largest meta-analyses of these diseases conducted in European populations to date (see Methods; Supplementary Table 3, Supplementary Data 2). We observed no association with the T1D PRS and increased risk of hypothyroidism or hyperthyroidism irAE (Fig. 2a, c). We found that a higher vitiligo PRS was associated with increased risk of hypothyroidism irAE (meta-analysis, *p* = 1.10 × 10^−6^; HR = 1.41, 95% CI 1.22–1.61 per unit normalized PRS) and hyperthyroidism irAE (meta-analysis, *p* = 0.0012; HR = 1.46, 95% CI 1.16–1.85 per unit normalized PRS) in patients treated with atezolizumab, but not in the control arms (Fig. 2a, c). Our results were consistent with the genetic correlation (*r*_g_ = 0.37; *p* = 5.57 × 10^−11^) between vitiligo and hypothyroidism as computed by LD score regression and emphasize the role of the immune system and autoimmunity during thyroid irAE.

A PRS represents a weighted linear sum of genetic variants that contribute to lifetime risk. Given that the environmental exposures that trigger thyroid autoimmunity or vitiligo in European populations over a lifetime are more varied than cancer treatment with atezolizumab, we hypothesized that only a subset of these lifetime risk variants contribute to risk for hypothyroidism irAE in atezolizumab treated patients. To test this, we used 3-fold cross validation to estimate the best sparseness penalty in a survival lasso model using the variants and coefficients from each PRS independently (see “Methods”). We then determined which variants were retained when a model was fit to all of the data using the estimated sparseness penalty. We found that 19 variants in the hypothyroidism PRS and 16 variants in the vitiligo PRS were assigned non-zero coefficients by lasso regression (Fig. 2d, Supplementary Fig. 11). We examined the transcription start sites (TSS) of genes within and near the credible sets (±500 kb from the ends) to which these variants belonged (Supplementary Data 4–5). We found that genes implicated in immune regulation and autoimmunity were enriched near these retained variants including *CTLA4, CBLB, PTPN22*, and *CD69* as well as known autoantigens tyrosinase (*TYR*) and thyroglobulin (*TG*). Lasso retained far fewer variants near genes implicated in thyroid development and function (e.g., *FOXE1* and *CAPZB*). Variants near *LPP*, a gene with no known immune function, were retained by lasso across both PRS. Further analysis of promoter capture Hi-C data and expression quantitative trait loci (eQTL) data indicated these variants likely effect *BCL6*, the lineage defining transcription factor for CD4^+^ T-follicular-helper (Tfh) cells (Supplementary Fig. 12)^32^.

### Genetic variation affects risk of thyroid irAE during anti-PD-L1 treatment

**Fig. 3:**
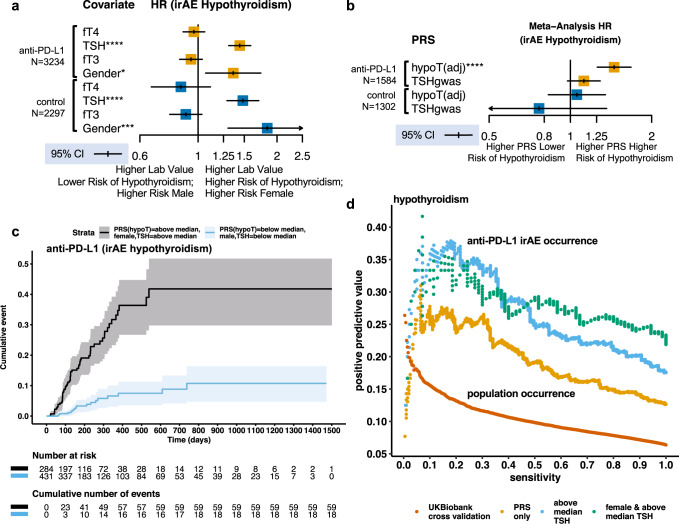
Genetic variation associated with lifetime risk of thyroid autoimmunity affects hypothyroidism irAE risk during atezolizumab treatment and acts independently of pre-treatment risk factors. **a** IPD meta-analysis assessing the association between hypothyroidism irAE and potential pre-treatment risk factors in a multivariable mixed effects Cox model fit to data from (*N* = 3234) atezolizumab and (*N* = 2297) standard of care treated cancer patients in the safety evaluable population with pre-treatment thyroid hormone measurements across the 7 clinical trials analyzed stratified across arms. Measurements were normalized across patients by normalization to the quantiles of a standard normal distribution and modeled as random effects. Point estimates and 95% CI for HR for hypothyroidism expressed in unit normalized hormone levels after fitting the model. TSH = pre-treatment measured thyroid-stimulating hormone; fT4 = free thyroxine; fT3 = free triiodothyronine. Gender is encoded as 1 = female and 0 = male. *p*-values for a two-sided Wald test that the log-HR is non-zero for fT4 *p* = 0.44, TSH *p* = 9.93 × 10^−14^, and gender *p* = 0.012 in atezolizumab treated patients and fT4 *p* = 0.25, TSH p = 3.26 × 10^−8^, and gender *p* = 0.00054 in standard of care treated patients. **b** Random effect point estimate and 95% CI for the IPD meta-analysis hazard ratio expressed in normalized unit PRS for the time to occurrence of hyperthyroidism irAE estimated using a mixed effect Cox model with genotype eigenvectors as fixed effect covariates in (*N* = 1584) atezolizumab and (*N* = 1302) standard of care treated European ancestry cancer patients across 7 clinical trials. TSHgwas uses a PRS constructed from a GWAS of TSH levels in individuals not receiving any medication for thyroid dysfunction. hypoT(adj) computes the association between a hypothyroidism irAE and the hypothyroidism PRS adjusted for measured pre-treatment TSH levels and gender using these as additional fixed effect covariates in the model. Meta-analysis *p*-values for a two-sided Wald test that the estimated log-HR is non-zero for hypoT(adj) *p* = 3.91 × 10^−7^ and TSHgwas *p* = 0.11 in atezolizumab treated patients and hypoT(adj) *p* = 0.66 and TSHgwas *p* = 0.36 in the standard of care treated patients stratified across trial arms. **c** Cumulative incidence plot comparing risk of hypothyroidism in atezolizumab patients with and without all of the pre-treatment risk factors identified for hypothyroidism irAE in atezolizumab treated cancer patients. **d** Positive predictive value and sensitivity (also known as precision and recall) for hypothyroidism irAE and population hypothyroidism occurrence across thresholds for the PRS in atezolizumab treated patients and estimated by 4-fold cross validation in the UK Biobank respectively. Curves were also generated for subgroups that have increasing incidence of hypothyroidism irAE. Meta-analysis: *p < 0.05, **p < 0.01, ***p < 0.001, ****p < 0.0001.

Genetic and environmental factors together may contribute to damage to the thyroid of patients over the course of their lifetime prior to cancer treatment. Hypothyroidism in human populations is more common in females than males, and patients may have differing levels of thyroid function^24^. We additionally considered whether these factors were relevant to risk of hypothyroidism irAE in atezolizumab treated patients. Within our cohort, TSH, free thyroxine (fT4), and free triiodothyronine (fT3) were measured in patients prior to treatment. We assessed whether these lab measures and gender were relevant to risk of hypothyroidism irAE during cancer treatment. Using a multivariable Cox model we found that measured pre-treatment TSH levels and gender, but not pre-treatment fT4 or fT3 levels, were independently associated with increased risk of hypothyroidism irAE, but not hyperthyroidism, in both the atezolizumab and the control arms (see “Methods”; Fig. 3a; Supplementary Fig. 13).

Given that measured pre-treatment TSH and gender were independently associated with risk of hypothyroidism irAE during atezolizumab treatment, this raised the possibility that the PRS and hypothyroidism irAE risk association we found might be explained by these factors. Genetic variation affecting thyroid function to set baseline TSH levels might contribute to risk of hypothyroidism irAE in atezolizumab treated patients. To address this possibility, we constructed a PRS using a quantitative GWAS of TSH levels from European individuals not receiving any medication for thyroid dysfunction^33^. In contrast to the hypothyroidism GWAS, enrichment in thyroid specific enhancers was clearly present, with no evidence of immune cell enrichment (Supplementary Figs. 6, 14). While the PRS was correlated with measured pre-treatment TSH levels (Spearman’s *r*_s_ = 0.228) in cancer patients, we observed no association with the TSH PRS and increased risk of atezolizumab-induced hypothyroidism (Fig. 3b). We also found a weaker correlation between the hypothyroidism PRS and measured pre-treatment TSH levels (Spearman’s r_s_ = 0.109) suggesting that damage to thyroid due to autoimmunity over a patient’s lifetime prior to cancer treatment may account for our findings. To address this additional possibility, we repeated our analysis of the association between the hypothyroidism PRS and hypothyroidism irAE including measured pre-treatment TSH levels and gender as covariates in our analysis. We found the association in atezolizumab treated patients was unchanged (HR = 1.45, 95% CI 1.25–1.67 per unit normalized PRS; Fig. 3b). Together, these additional analyses indicate that the hypothyroidism PRS and irAE risk association is not explained by thyroid function prior to atezolizumab treatment or by gender, but arises from the effect of genetic variation during the course of atezolizumab treatment.

As we identified several independent risk factors associated with hypothyroidism irAE in atezolizumab treated patients, we explored whether these factors could be combined to identify sub-groups at high or low risk and to develop a pre-treatment predictor. Combining each independent risk factor, we found that risk of these irAE was 6.85 (95% CI 3.49–13.44) times higher in female anti-PD-L1 treated patients with above-median hypothyroidism PRS and above-median baseline TSH levels as compared to male anti-PD-L1 treated patients with below-median PRS and below-median baseline TSH levels (Fig. 3c). To evaluate these factors as pre-treatment predictors of hypothyroidism irAE, we next quantified positive predictive value (PPV) and sensitivity (precision and recall) with which the hypothyroidism PRS correctly predicted hypothyroidism irAE occurrence at varying thresholds of the PRS in atezolizumab treated patients. We used these metrics due to the large imbalance between the positive and negative cases in these data. We also considered these properties in patient subgroups with higher incidence, as delineated by an above-median baseline TSH and additionally by gender (Fig. 3d). We compared these curves to a 4-fold cross validation estimate of the PPV and sensitivity for predicting population hypothyroidism cases by PRS in the UK Biobank (see “Methods”). Although the PRS had a PPV in atezolizumab treated patients that was higher than its population PPV, it did not achieve near perfect prediction even at a high cutoff with low sensitivity.

### Hypothyroidism PRS is associated with OS in anti-PD-L1 treated TNBC patients

**Fig. 4:**
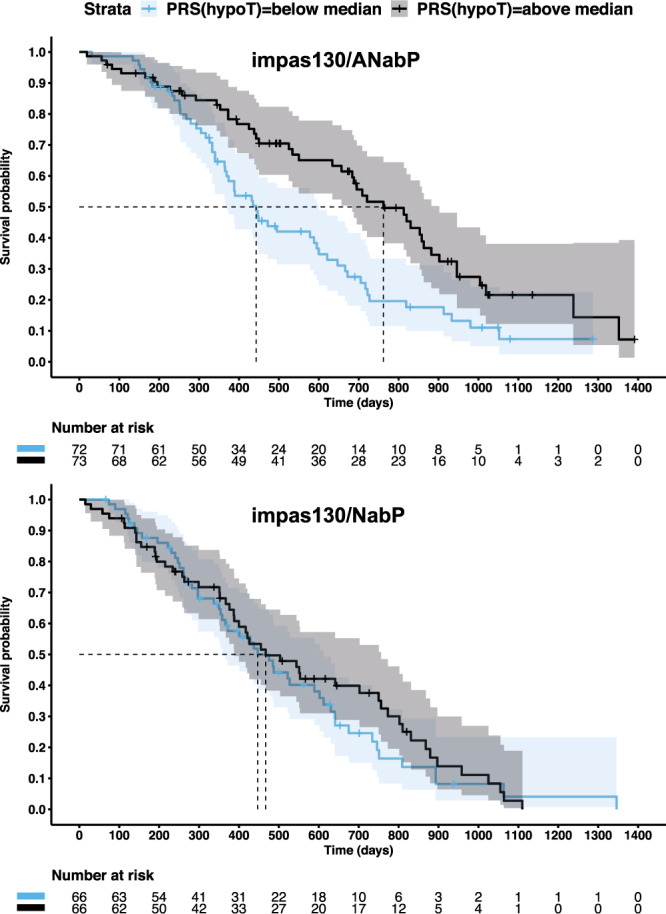
Polygenic risk for hypothyroidism is associated with lower risk of death in European ancestry TNBC patients treated with atezolizumab and nab-paclitaxel. Kaplan–Meier (KM) plot for OS for triple-negative breast cancer (TNBC) patients of European ancestry treated with atezolizumab plus nab-paclitaxel (top) and placebo plus nab-paclitaxel (bottom) from IMpassion130. Patients were split into two groups on the basis of median split of the hypothyroidism PRS (hypoT) across all European ancestry IMpassion130 patients with germline genetic data. Censoring events are denoted by vertical dashes. Dashed horizontal and vertical lines designate the median survival time. Shaded regions provide the 95% confidence intervals. impas130 = IMpassion130. A = atezolizumab; NabP = Nab-paclitaxel.

The low precision of the hypothyroidism PRS as a predictor for hypothyroidism irAE further limits its precision and sensitivity as predictor for OS. Both high precision and sensitivity is needed to recapitulate the association between the event occurrence and OS we previously observed (Fig. 1b). Given that we studied data across cancers, an exception to this observation might reflect the importance of genetic variation associated with lifetime risk for thyroid autoimmunity within the cancer indication. Therefore, we assessed whether the hypothyroidism PRS was associated directly with outcome measures within each of the trial arms we analyzed. Consistent with its limited precision and sensitivity, the hypothyroidism PRS was not associated with OS, PFS, or tumor response across most trial arms (Supplementary Fig. 15). However, we identified one notable exception, a positive association between the hypothyroidism PRS and lower risk of death in TNBC patients in the atezolizumab plus nab-paclitaxel arm of IMpassion130. The corresponding p-value met the cutoff for Bonferroni significance, accounting for the number of outcome measures and trial arms we tested (*p* = 5.06 × 10^−5^, HR = 0.62, 95% CI 0.49–0.78 per unit normalized PRS). We did not observe an association (*p* = 0.17, HR = 0.86, 95% CI 0.69–1.07 per unit normalized PRS) in the placebo plus nab-paclitaxel arm of this trial (Fig. 4). We confirmed that the association we observed in the atezolizumab arm was robust to inclusion of PD-L1 positivity in the tumor as a covariate, indicating the PRS was independently associated with survival in this trial (adjusted HR = 0.65, 95% CI 0.51–0.83 per unit normalized PRS, Supplementary Fig. 16).

TNBC varies in prevalence across ethnicities with higher prevalence in African Americans^34^. However, our hypothyroidism PRS was constructed using data from Europeans. Therefore, we assessed whether the PRS associations we observed in atezolizumab treated patients were transferable across ethnicities. We examined hypothyroidism irAE data from 312 atezolizumab treated patients from the trials we studied here that did not meet our cutoff for European ancestry and were excluded from our analysis (Supplementary Fig. 8). In this set of patients, we found no evidence that the UK Biobank derived hypothyroidism PRS was associated with risk of hypothyroidism irAE (meta-analysis, *p* = 0.09; HR = 1.43, 95% CI 0.95–2.18 per unit normalized PRS; Supplementary Fig. 17). Within this set of non-European patients, we also found no evidence of an association between the hypothyroidism PRS and OS in 93 atezolizumab treated TNBC patients from the IMpassion130 trial (*p* = 0.53, HR = 1.1, 95% CI 0.81–1.49) (Supplementary Fig. 18). Consistent with prior studies that have shown that PRS have limited trans-ethnic transferability, our findings, at present, are limited to cancer patients of European ancestry^35^.

## Discussion

The concept of a cancer-immune set point considers not only the immune profile of a tumor, but also the state of patient’s immune system to explain why some immunotherapy-treated patients mount effective and safe anti-tumor responses and others fail to respond or develop immune toxicities. Our study focused on atezolizumab-induced hypothyroidism irAE, which are noteworthy given that we show they are common and associated with longer patient survival across cancers and chemotherapy combinations. PRS derived from a GWAS of hypothyroidism or vitiligo cases and controls in European populations quantify one dimension of a patient’s cancer-immune set point: lifetime genetic susceptibility for tissue-specific autoimmunity. We find that this lifetime genetic susceptibility is correlated with risk of hypothyroidism irAE during the shorter duration of atezolizumab treatment. This implies genetic mechanisms that contribute to thyroid autoimmunity and vitiligo over a lifetime are magnified and accelerated to increase risk of hypothyroidism irAE during cancer treatment with atezolizumab.

Derived from loci in the genome, PRS provide insight into the mechanisms of hypothyroidism irAE. Using cross validation and survival lasso, we found that a subset of variants in the hypothyroidism and vitiligo PRS were retained by the regression model to explain variation in hypothyroidism irAE risk in atezolizumab treated patients. One of the most intriguing loci selected by lasso from both the hypothyroidism and vitiligo PRS was in the intron of the *LPP* gene. Both eQTL evidence and promoter capture Hi-C data indicate that this locus affects *BCL6*, the lineage defining transcription factor for Tfh cells, whose TSS was 692 kb away from the credible set. Given that PD-1 is a surface marker of Tfh cells, we hypothesize that atezolizumab disrupts critical interactions required for B-cell maturation and antibody affinity to contribute to irAE risk^32^. The variants retained also included the extensively studied R620W (rs2476601) variant in *PTPN22* highlighting the role of T-cell and B-cell receptor signaling^36^. Although this approach has important limitations, as demonstrated by the variant in the intron of *LPP*, examining the genes near credible sets to which retained variant belongs also highlighted genes involved in T-cell priming (*CTLA4*) and activation (*CD69*). Given that these retained variants contribute to inter-individual variation in a phenotype arising due to atezolizumab treatment, our study introduces an approach to identify pathways and genes modulated during PD-1 blockade.

Genetic susceptibility to thyroid autoimmunity may translate into survival benefit in atezolizumab plus nab-paclitaxel treated TNBC patients. Although this finding requires further validation, it raises hypotheses for investigation. Evidence of immune cross reactivity has been found in PD-1 checkpoint inhibitor myocarditis where T-cell clones in the myocardium at autopsy where the same T-cell clones in the patient’s tumor^37^. Thyroid auto-antibodies have been associated, in some contexts, with better breast cancer prognosis^38^. Immune cells cross reactive to antigens in both breast and thyroid, both glandular tissues, could explain the association between the PRS and OS in atezolizumab plus nab-paclitaxel treated TNBC patients. Even in the absence of cross reactivity, immune responses mediated by Tfh cells and B-cells might play a significant role in extending TNBC patient survival. Investigation of these hypotheses could provide avenues for therapeutic strategies and approaches to stratify patients in TNBC.

Our findings have implications for clinical management of cancer patients treated with PD-1 checkpoint inhibitors and for the use PRS more broadly. When combined with additional risk factors, the hypothyroidism PRS identifies subgroups with a > 6-fold difference in risk for atezolizumab-induced hypothyroidism irAE. As most hypothyroidism irAE occur within the first year of treatment with atezolizumab, this application of a PRS has greater potential for meaningful impact on individual patients than use of PRS to report lifetime risk. Yet, the use of PRS in clinical settings has an important limitation. At present the trans-ethnic transferability of PRS is limited^35^. Non-European populations are underrepresented in GWAS and correspondingly in PRS^39^. Algorithms that enhance the trans-ethnic transferability of PRS remain an active area of research. These important challenges must be overcome to ensure all patients benefit from the information provided by PRS.

In summary, hypothyroidism irAE arise as a consequence of the systemic immune response to PD-1 blockade. We demonstrate that genetic variation associated with lifetime risk of thyroid autoimmunity shapes this systemic immune response to contribute to irAE susceptibility during atezolizumab treatment. Further study of the mechanisms that underlie this interaction may provide a basis for informing therapeutic strategies that can mitigate risk of immune toxicity and improve the efficacy of PD-1 checkpoint inhibitors.

## Methods

### Patient cohorts

We conducted a retrospective meta-analysis analysis of immune-related adverse events (irAE) using individual participant data from 7 previously completed randomized controlled trials. Detailed clinical trial results have been previously reported, and the clinical trial protocols have been provided as supplementary materials in the original study publications^15–22^. All of the trials were sponsored by F. Hoffmann–La Roche/Genentech. The sponsor provided the study drugs and collaborated with the investigators across countries and study sites on the clinical trial and collection of the data. Each trial was conducted in accordance with the International Conference on Harmonization Good Clinical Practice guidelines and with the principles of the Declaration of Helsinki. An independent data monitoring committee reviewed safety data from these studies.

All patients provided informed consent for the main study. A subset of patients signed an optional Research Biosample Repository (RBR) Informed Consent Form (ICF) to provide whole blood samples for future research. By signing the optional RBR ICF, patients provided informed consent for study of inherited and non-inherited genetic variation from these whole blood samples. As illustrated in the consort diagram in Supplementary Fig. 8 whole-genome sequencing data was collected from whole blood only from patients that signed the optional RBR ICF. Ethics Committees (EC) and Institutional Review Boards (IRB) at each study site for each clinical trial approved the clinical trial protocol, the main study ICF, and the RBR ICF. The EC and IRB for each clinical trial and study site are provided in Supplementary Note 1.

### Endocrinopathies analyzed

irAE were defined on the basis of an adverse events of special interest (AESI) strategy uniformly applied to the clinical trials we studied here. The original clinical trial protocols provide details on this methodology. We focused on the following endocrinopathies that were captured by the AESI strategy: hypothyroidism, hyperthyroidism, adrenal insufficiency, type 1 diabetes mellitus, and hypophysitis. We note that patients with active autoimmune disease were excluded from the clinical trials we analyzed - in accordance with each study protocol.

### Frequency of thyroid function lab testing

In all studies, endocrine irAE were captured on the basis of clinical symptoms and investigator lab tests as they appeared during treatment. Study protocols included provisions for thyroid hormone testing at regular intervals during treatment that varied on a per study basis. IMvigor211 initially conducted lab tests for TSH, fT4, and fT3 levels at screening and treatment discontinuation, but a protocol amendment changed this to monitor at cycle 5 and every 4 cycles thereafter. The IMpower studies measured TSH, fT4, and fT3 levels at screening, treatment discontinuation, Cycle 1, and every fourth cycle thereafter. IMmotion151 and IMpassion130 conducted thyroid labs at screening, treatment discontinuation, and every two cycles (starting at Cycle 2).

### Time-dependent covariate to overall survival association meta-analysis

To assess whether there exists an association between occurrence of an irAE and overall survival (OS) we used a time-dependent covariate. The time-dependent covariate was set to zero before the irAE onset and then to one after the irAE to estimate the association between the occurrence of irAE and OS. The covariate was set to zero for patients that did not experience the irAE. We constructed the time-dependent covariate using the tmerge function in the survival package in R. This generated interval data for use in a Cox proportional hazards model. Across the trial arms, we then performed an individual participant data meta-analysis using the coxme package in R. Our model used a differing baseline hazard for each trial arm and also allowed for heterogeneity of the hazard ratio across the trial arms by use of a random effect term as indicated by the following R formula: *Surv(tstart, tstop, OS.tv) ~ irAE + (irAE|trial.arm) + strata(trial.arm)* where irAE corresponds to the time-dependent covariate, trial.arm stratifies and groups according to trial arm, and *tstart* and *tstop* designate an interval and *OS.tv* is set to 1 if a death event occurred at the end of the interval and 0 if censored or if the interval preceded the irAE.

### Construction of a UK Biobank linkage disequilibrium (LD) reference panel

We removed individuals that were not used in the PCA calculation, that were outliers for heterozygosity or missing rate, that had excess (ten or more) relatives identified, and had evidence of sex chromosome aneuploidy. We then selected individuals that self-reported “British”, “Irish”, “White”, or “Any other white background” ancestry. We randomly sampled 10,000 individuals from this cohort to construct an LD reference panel. We recomputed the per variant MAF and imputation INFO scores in this cohort and removed variants with MAF < 0.001. We kept variants meeting the following criterial for INFO score at several levels of MAF: Info > 0.3 for MAF > 0.03; Info > 0.6 for MAF 0.01–0.03; Info > 0.8 for MAF 0.005–0.01; Info > 0.9 for MAF 0.001–0.005. These filters left a total of 12.5 million variants in the LD reference panel. This LD reference panel was used in all subsequent analyses as a European LD reference panel.

### Fine mapping

We adapted an approach used by the DIAGRAM consortium in Type-2 diabetes^40^. As detailed below, we used GCTA-COJO to perform forward selection using approximate conditional analyses to detect distinct association signals^41^. GCTA-COJO performs approximate conditional analysis using GWAS summary statistics and an LD reference panel. We used the following algorithm to identify a set of independent signals, represented by a set of conditioning SNPs, within a given associated locus:

1. Identify a lead SNP from the unconditional meta-analysis and define locus ±500 kb around the lead SNP. Merge overlapping loci into one locus.
2. Condition out the lead SNP using GCTA-COJO.
3. If top SNP after conditional meta-analysis meets the genome-wide significance threshold (5 × 10^−8^) then repeat step 2, conditioning out the original lead SNP and the new top SNP.
4. Continue steps 2 and 3—effectively forward selection—until the residual conditional meta-analysis does not meet the significance threshold.
5. This procedure will provide a set of “conditioning SNPs” at a given locus.

We then constructed “Wakefield” credible sets from the independent signals represented by a set of conditioning SNPs^42^. The algorithm conditioned out all but one SNP to obtain conditionally independent signals and corresponding summary stats which were then used to construct credible sets. To illustrate the algorithm for constructing credible sets, we provide an example for three conditioning SNPs: rsA, rsB, and rsC

1. To get the conditional association signal for rsA, condition out both rsB and rsC using GCTA-COJO.
2. Using the resulting conditional summary stats, compute the per variant approximate Bayes factor (ABF) ${{\rm{ABF}}}$ where $\beta$ corresponds to the log odds ratio and $\sigma$ the standard error after conditioning out all but the focal variant.
  1 $$r=\frac{W}{W+{\sigma }^{2}}$$
  2 $$z=\frac{\beta }{\sigma }$$
  3 $${{{\rm{ABF}}}}_{{H}_{0}}=\frac{1}{\sqrt{1-r}}{\exp }\left\{-\frac{{z}^{2}}{2}r\right\}$$
  4 $${{\rm{ABF}}}=\frac{1}{{{{\rm{ABF}}}}_{{H}_{0}}}$$
  here, we set $W=0.04$ which assumes that the 97.5% point of the prior is 1.48, that is, the prior probability that the odds ratio is greater than the 97.5% point is 0.025.
3. Compute the posterior probably of association (PPA) values for variant $i$ by normalizing by all variants in the region by the sum of the ${{\rm{ABF}}}$ values
  5 $${{{\rm{PPA}}}}_{i}=\frac{{{{\rm{ABF}}}}_{i}}{\mathop{\sum}\limits_{k}{{{\rm{ABF}}}}_{k}}$$
  Construct the 99% Wakefield credible set was by ordering all variants in descending order of their PPA including the ordered variants into the set until the cumulative PPA is ≥0.99.
4. Repeat same process to obtain association signal for rsB by conditioning out rsA and rsC, and association signal for rsC by conditioning out rsA and rsB. After each conditioning step, compute the PPA and Wakefield credible steps.

We applied this algorithm to the hypothyroidism, T1D, and vitiligo summary statistics. Variants with the highest PPA within each credible set and their corresponding conditional effect sizes were used to compute a polygenic risk score as detailed below.

### Whole-genome sequencing and sample/variant QC of atezolizumab trial cohort

Genomic DNA was extracted from blood samples using the DNA Blood400 kit (Chemagic) and eluted in 50 μL Elution Buffer (EB, Qiagen). DNA was sheered (Covaris LE220) and sequencing libraries were prepared using the TruSeq Nano DNA HT kit (Illumina Inc.). All sequencing data was checked for concordance with SNP fingerprint data collected before sequencing. 150 bp paired-end whole-genome sequencing (WGS) data was generated to an average read depth of 30x using the HiSeq platform (Illumina X10, San Diego, CA, USA).

Reads were aligned using the functionally equivalent BAM (FEB) pipeline. Samples were joint genotyped using Sentieon GATK. Only variants flagged as PASS and genotype calls with GQ > 20 were used. After application of the GQ filter, variants with genotype call missing rate of >0.1 were removed. Multi-allelic sites were handled by keeping only calls for the two most common alleles, all other calls were set to missing. Common variants with MAF > 0.01 in this cohort were extracted. Samples were removed if they had a high within-sample missing rate of >0.1. Samples were then merged with 1000 Genomes samples, and LD pruned. ADMIXTURE v1.23 was used to estimate ancestry in the 5 major populations using supervised mode. Samples with >0.7 European (EUR) ancestry were extracted and analyzed for heterozygosity outliers by estimating the per sample F inbreeding coefficient. EUR samples with an F statistic more than 5 standard deviations from the mean were removed. EUR samples were then analyzed for relatedness using the KING method implemented in the SNPRelate R package. Sample pairs with Pr(no allele shared), k0 < 0.4, were identified and the sample with the least missing variant calls was kept and the other removed. We then performed PCA using the implementation in the SNPRelate package. Five rounds of PCA outlier removal iterations were performed removing samples that were >6 standard deviations from the top 10 eigenvectors at each iteration. The final PCA was then performed to compute 5 eigenvectors that were subsequently used to account for any remaining population stratification.

Using this final EUR cohort with missing rate, heterozygosity, relatedness, and PCA outlier samples removed, we performed variant level QC. We analyzed heterozygous calls for variants for evidence of allele imbalance by summing counts of each of the alleles at these het calls using the AD value in the VCF file and removing variants with an allele balance of <0.3 or >0.7 or a binomial test *p* < 5 × 10^−8^. Variants were also analyzed for violation of Hardy Weinberg equilibrium at *p* < 5 × 10^−8^ and any variants with MAF < 0.001 were removed. In total, 14.3 million variants were left after these filtering steps.

### Harmonization of summary statistics with LD reference panel

Each of the summary statistics were harmonized with the LD reference panel as well as the atezolizumab trial whole genome sequencing data by using genomic position. The effect and non-effect alleles were also required to match between the LD reference, the atezolizumab trial data, and the summary statistics. Variants with strand ambiguity (A/T or C/G genotypes) were removed. The UCSC genome browser chain files were used to lift any hg19 coordinate systems to hg38 as necessary using the rtracklayer package in R. All variants in the MHC region (hg38, chr6: 28510120-33480577) were removed due to the complex LD in this region for both fine mapping and subsequently construction of a PRS. The summary stats after this harmonization are provided for download from the links provided in Supplementary Table 3.

### Construction and computation of polygenic risk scores

For each independent signal’s 99% credible set, we used the variant with the highest PPA in the polygenic risk score. The score used effect sizes from the conditional summary stats obtained after conditioning out all but one of the conditioning SNPs as detailed above. The PRS was computed as follows:

7 $$\hat{S}={\sum }_{i=1}^{M}{\beta }_{i}\cdot {G}_{i}$$

where $M$ is the number of independent signals in the GWAS after fine mapping and ${\beta }_{i}$ corresponds to the conditional effect size for the variant with the highest PPA for $i$th signal and ${G}_{i}=\left\{{\mathrm{0,1,2}}\right\}$ corresponds to the number of copies of the risk allele. PRSs were quantile normalized to the quantiles of a standard normal distribution to allow comparison across GWAS.

### Construction of an LDpred2-auto hypothyroidism PRS

After the harmonization steps above, we restricted the number of variants to those in the HapMap3 project on the basis of rsid match to a UK Biobank provided rsid. In total, 1,099,649 HapMap3 variants were used. Following the methods detailed in the LDpred2 vignette, we interpolated genomic positions (in bp) to genetic positions (in cM). We next computed the correlation matrices chromosome-wise using a window size of 3 cM. We then applied LDpred2-auto running 24 separate Gibb’s samplers the following initial values for the fraction of causal variants *p*: 1e−04, 1e−04, 2e−04, 3e−04, 5e−04, 7e−04, 0.001, 0.0015, 0.0022, 0.0032, 0.0047, 0.0069, 0.0101, 0.0149, 0.0219, 0.0322, 0.0473, 0.0695, 0.1021, 0.1501, 0.2206, 0.3241, 0.4763, 0.7. The initial value for heritability *h*^2^ was set to the per-chromosome estimate of heritability obtained by LD-score regression. Each run of LDpred2-auto Gibb’s sampler generated a per-chromosome estimated value for *h*^2^ and *p* as well as set of shrunk beta coefficients. We inspected the paths of the Gibb’s samplers for *p* and *h*^2^ and found that the majority of Gibb’s samplers converged to the same values for these parameters. Deviating from the recommendations of the vignette, we computed the median value of the estimated fraction of causal variants *p* across all the 24 Gibb’s samplers. We selected the shrunk beta coefficients associated to this median value for *p*. This approach was applied chromosome-wise. Last, we combined the selected shrunk beta coefficients genome-wide and computed the PRS across 1,099,649 HapMap variants with these coefficients within the atezolizumab clinical trial cohort as described in the previous section.

### PRS and irAE meta-analysis across trial arms

Since we had access to individual participant data across trials, we used a one-step approach to conduct a meta-analysis. For time to event data, we used a mixed effects Cox model that allowed for a differing baseline hazard per trial arm using the coxme package in R. Our analysis also accounted 5 genotype eigenvectors to account for remaining population stratification. The meta-analysis p-value corresponded a two-sided Wald test for a non-zero coefficient on the PRS term in the following coxme model: *Surv(irAE.time, irAE.occured)* *~* *PRS* *+* *(PRS* | *trial.arm)* *+* *EV.1* *+* *EV.2* *+* *EV.3* *+* *EV.4* *+* *EV.5* *+* *strata(trial.arm)*. By modeling a random effect on PRS, the model allowed for some heterogeneity among the trial arm specific effect sizes.

### Identification of important PRS variants by survival lasso regression

In order to identify which subset of variants contributed substantially to the association between PRS and hypothyroidism irAE, we created an $N\times P$ data matrix for $i=1\ldots N$ atezolizumab treated patients and $p=1\ldots P$ variants from the polygenic risk score. For a given individual $i$ in the data matrix and variant $p$, we set the element of the data matrix to number of copies of the risk allele carried by individual $i$ multiplied by the conditional effect size of variant $p$. We additionally added genotype eigenvectors as additional columns to the data matrix. We then fit this data matrix using a survival lasso model where the coefficients of the model were constrained to be $\ge 0$ with the exception of the coefficients associated with the genotype eigenvectors. We also excluded the genotype eigenvectors from the ${l}_{1}$ penalty allowing the model to adjust for these covariates independently. We used 3-fold cross validation to estimate the best ${l}_{1}$ penalty parameter in a survival lasso model for time of hypothyroidism irAE occurrence. Cross validation was run 100 times and the average obtained across runs. This run average best ${l}_{1}$ penalty parameter was used to determined which variants were retained when a model was fit to all of the data. To estimate the relative importance of retained variants, the resulting non-zero lasso coefficients for the variants were multiplied by the conditional effect sizes provided by fine mapping, thus providing re-weighted coefficients after the model was fit to the time to hypothyroidism irAE data. The resulting absolute values were normalized to sum to one. We confirmed that this approach did not assign any non-zero coefficients to variants for patients in the control arms that developed hypothyroidism. Candidate genes within and near the credible sets were identified using transcription start sites of APPRIS principal isoforms in the GENCODE v32 gene annotations.

### Testing the association between thyroid irAE and hormone levels and gender

We used an individual participant data meta-analysis approach to determine whether there exists an association between the risk of thyroid irAE and the following factors: thyroid hormone levels TSH, ft4, and fT3 at baseline and gender. TSH, ft4, and fT3 levels were normalized to the quantiles of a standard normal (using qqnorm function in R). The normalization allowed comparison across hormone levels. We encoded the gender variable as female = 1 and male = 0. To conduct the meta-analysis, we used the coxme package in R. The model allowed for a random effect on each of the hormone levels and gender, allowing for differing effect sizes across each trial arm. The model also allowed for a differing baseline hazard across each trial arm. The 95% confidence intervals around the hazard ratios expressed in unit normalized hormone levels were reported.

### Cross validation estimation of PRS PPV, sensitivity, and effect size in the UK Biobank

To estimate the PPV and sensitivity (precision and recall) curve for a hypothyroidism PRS as applied to population occurrence of hypothyroidism, we performed 4-fold cross validation in the UK Biobank. Specifically, we conducted a hypothyroidism GWAS on a training fold using both ICD code diagnosed and self-reported cases using SAIGE^28^. Then, we created a PRS using summary statistics from this training fold using GCTA-COJO forward selection and by selecting the max PPA variant to include in the PRS—the identical algorithm we used to create the PRS used in our study of cancer patients from the atezolizumab trials. Then, we applied the PRS to the test fold from the UK Biobank and quantile normalized the PRS values to the quantiles of a standard normal in the test fold so they were comparable across folds. We combined the test folds and estimated the positive predictive value (PPV) and sensitivity (precision and recall) curve for varying thresholds on the PRS where above threshold designated predicted case status. We estimated effect size between cases and controls by computing the mean PRS value for cases and the mean PRS value for controls within each test fold. The effect size was estimated as the absolute value of the difference between the means divided by the standard deviation of the PRS values computed in the test folds. The cross validated effect size estimate was the average of the within test fold estimated effect sizes.

## Supplementary information

Supplementary Information

Supplementary Data 1

Supplementary Data 2

Supplementary Data 3

Supplementary Data 4

Supplementary Data 5

## Data Availability

Individual level UK Biobank data is available by application at https://www.ukbiobank.ac.uk/. UK Biobank hypothyroidism GWAS summary statistics used for PRS construction in this study can be accessed, viewed, and downloaded in full here: https://my.locuszoom.org/gwas/552910/. Variants and coefficients used to compute polygenic risk scores in this study are provided as Supplementary Data 3–4. The PRS validated in this study can be programmatically accessed and annotated with additional meta-data in the PGS Catalog at accession PGP000164. Qualified researchers may request access to individual patient data used in this study through Roche’s data sharing platforms in accordance with the Global Policy on Sharing of Clinical Study Information: http://www.roche.com/research_and_development/who_we_are_how_we_work/clinical_trials/our_commitment_to_data_sharing.htm. To ensure compliance with legal, data retention, and patient confidentiality obligations in the informed consent forms (ICF), the whole-genome sequencing data collected (in VCF or BAM/FASTQ formats) cannot be hosted on a public, controlled access repository and will be made available to individual requestors on completion of a data sharing agreement with Roche/Genentech. Requests for access to whole-genome sequencing data should be made to the corresponding authors by email at Zia Khan (khanz12@gene.com) or G. Scott Chandler (g_scott.chandler@roche.com). The planned research with the requested data will be reviewed by the Roche Pharma Repository Governance Committee to assess its scientific merit and to ensure it is in scope of the ICF approved locally at each study site.
