# Supplementary material for: Genetic variation associated with thyroid autoimmunity shapes the systemic immune response to PD-1 checkpoint blockade
Source: Nat Commun. 2021 Jun 7;12:3355. doi: 10.1038/s41467-021-23661-4 (PMC8184890; doi:10.1038/s41467-021-23661-4)
Supplement: Supplementary file 1 — Supplementary Information [file 41467_2021_23661_MOESM1_ESM.pdf]

Supplementary Information for *Genetic variation associated with thyroid autoimmunity shapes the systemic immune response to PD-1 checkpoint blockade*

Supplementary Tables

Supplementary Table 1. Atezolizumab trials analyzed in this study. NSCLC: non-small cell lung cancer; SCLC: small cell lung cancer; RCC: renal cell carcinoma; TNBC = triple negative breast cancer; 1L = first line, 2L = second line

Trial	Abbreviation	Indication	Citation	NCT Number
IMvigor211	imv211	2L metastatic urothelial carcinoma	[1]	NCT02302807
IMpower150	imp150	1L non-squamous NSCLC	[2]	NCT02366143
IMpower131	imp131	1L squamous NSCLC	[3]	NCT02367794
IMpower130	imp130	1L non-squamous NSCLC	[4]	NCT02367781
IMpower133	imp133	SCLC	[5]	NCT02763579
IMmotion151	imm151	RCC	[6]	NCT02420821
IMpassion130	impas130	TNBC	[7,8]	NCT02425891

Supplementary Table 2. Number of patients in the safety evaluable population. Number of patients that provided informed consent for genetic data collection, were of European (EUR) ancestry, and met our genotype and population QC filters separated by trial and arm (see Methods).

Trial	Arm Abbreviation	N (safety)	N(consent)	N(EUR)	N(EUR QC)
IMvigor211	Atezo	459	243	228	224
IMvigor211	Chemo	443	236	212	204
IMpower150	ABCP	393	249	210	200
IMpower150	ACP	400	267	229	223
IMpower150	BCP	394	236	185	178
IMpower131	ACNabP	334	192	161	155
IMpower131	ACP	332	188	165	158
IMpower131	CNabP	334	193	160	155
IMpower130	ACNabP	473	226	204	200
IMpower130	CNabP	232	116	103	103
IMpower133	ACE	250	96	77	76
IMpower133	CE	244	85	71	71
IMpassion130	ANabP	460	259	166	145
IMpassion130	NabP	430	230	142	132
IMmotion151	AB	451	252	214	203
IMmotion151	SUN	446	218	194	189

Abbreviations: Atezo=atezolizumab monotherapy; A=atezolizumab; C=carboplatin; P=paclitaxel; NabP=Nab-paclitaxel; B=bevacizumab; SUN=sunitinib; E=etoposide; Chemo=taxanes or vinflunine; N(EUR) designates individuals with ADMIXTURE EUR ancestry coefficients of > 0.7, N(EUR QC) designates individuals kept after population and genotype level QC filters.

Supplementary Table 3. GWAS Summary Statistics Analyzed in this Study

Abbreviation	GWAS	Cases/Controls	Citation	URLs
TSHgwas	TSH levels, excluding patients receiving medication for thyroid dysfunction	54288/NA	[9]	https://transfer.sysepi.medizin.uni-greifswald.de/thyroidomics/datasets/
hypoT	ICD Code + Self Reported Hypothyroidism UK Biobank	25072/383887	[10]	PheCode_244_and_selfreport_SAIGE_MACge20.txt.vcf.gz.lifted.gz obtained from: https://console.cloud.google.com/storage/browser/finngen-production-library-green/finngen_R4/finngen_R4_analysis_data/ukbb_meta/ukbb_summary_stats GWAS results after harmonization with whole genome sequencing data are also provided here: https://my.locuszoom.org/gwas/552910/?token=4ce42592a2334fdd8d129af8573c6935 and here https://my.locuszoom.org/gwas/552910/
T1D	Type-1 Diabetes Meta-analysis	5913/8828	[11]	https://datadryad.org/stash/dataset/doi:10.5061/dryad.ns8q3
VIT	Vitiligo GWAS	4680/39586	[12]	ftp://ftp.ebi.ac.uk/pub/databases/gwas/summary_statisti cs/JinY_27723757_GCST004785

NA = not applicable

The hypothyroidism GWAS was conducted using SAIGE[13]. The genomic inflation factor for the hypoT GWAS was λ_{gc} = 1.192. As λ_{gc} scales with sample size, we computed the inflation factor for an equivalent study of 1000 cases and 1000 controls λ_{1000}=1.004 confirming no significant test statistic inflation[14].

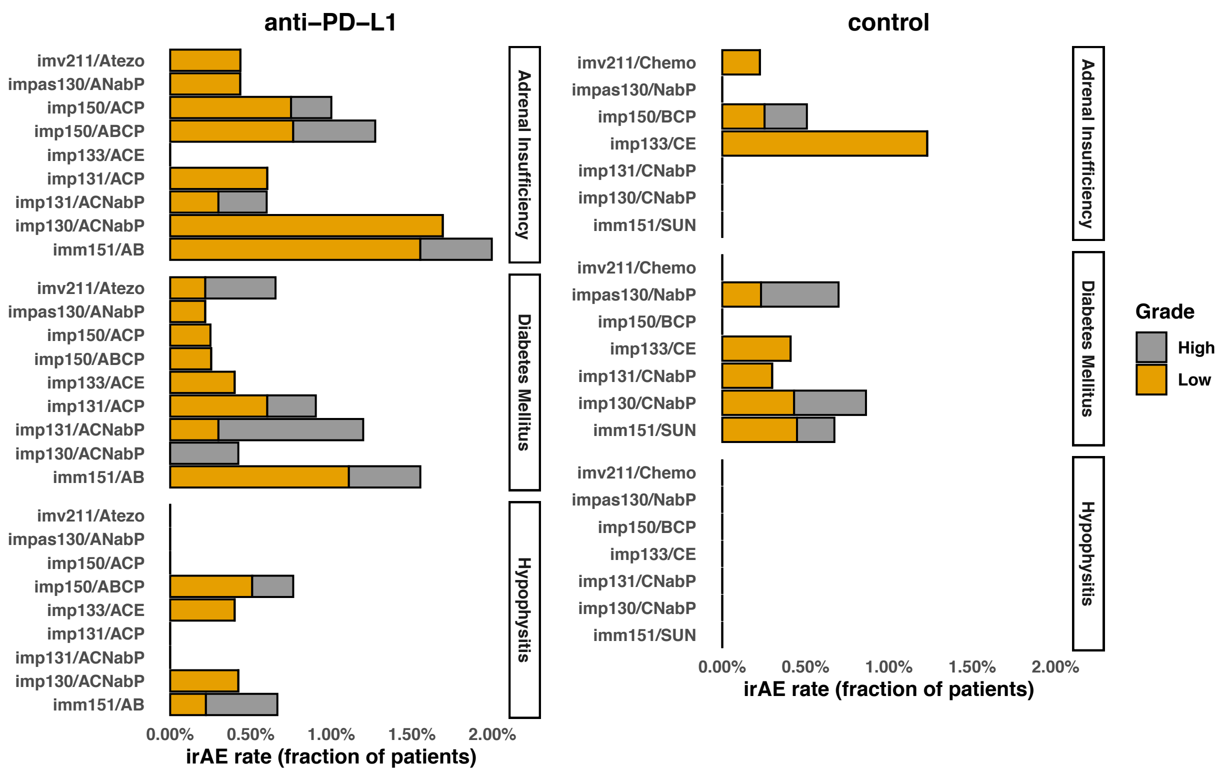

Supplementary Figure 1. Fraction of patients that developed rare endocrine irAE across trials and trial arms analyzed. Fractions were were computed in the entire safety evaluable population. CTCAE High: ≥ Grade 3 and Low: Grade 1-2.

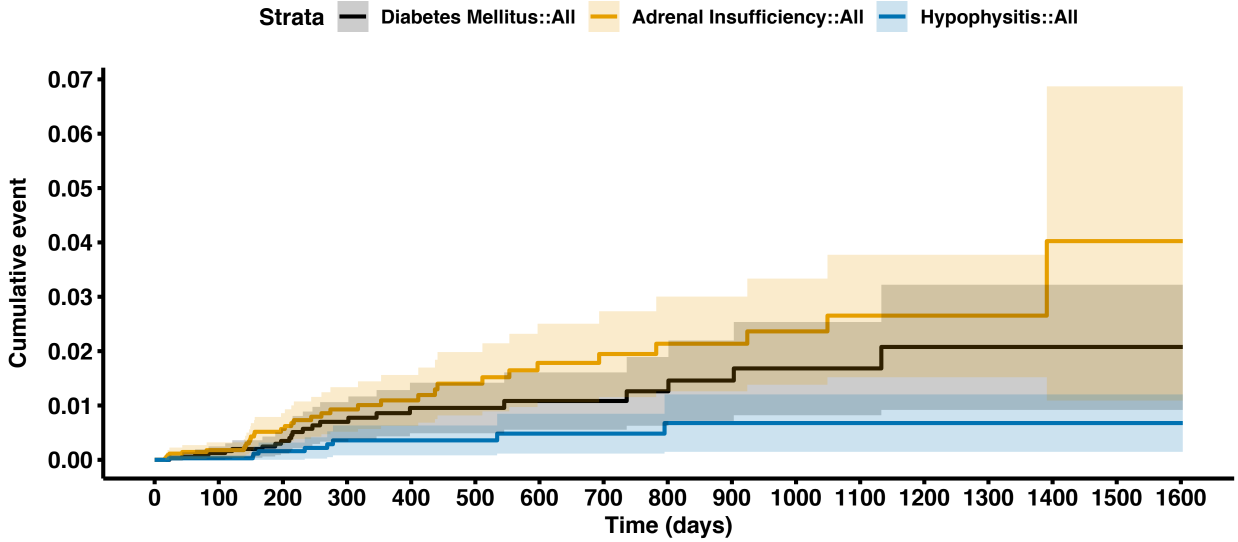

Number at risk

3552	2768	1876	1321	1027	846	711	600	501	443	374	286	199	129	56	13	1
3552	2768	1878	1324	1031	847	711	599	502	446	377	290	206	135	60	17	1
3552	2771	1882	1329	1036	852	717	606	507	450	380	293	206	134	60	16	1

Cumulative number of events

0	4	9	15	18	18	19	19	20	21	22	22	23	23	23	23	23
0	6	15	21	23	26	29	30	31	31	32	33	33	33	34	34	34
0	1	4	7	7	7	8	8	9	9	9	9	9	9	9	9	9

Supplementary Figure 2. Incidence plot for rare irAE across all trials for all safety evaluable patients. Shaded regions show the 95% confidence intervals.

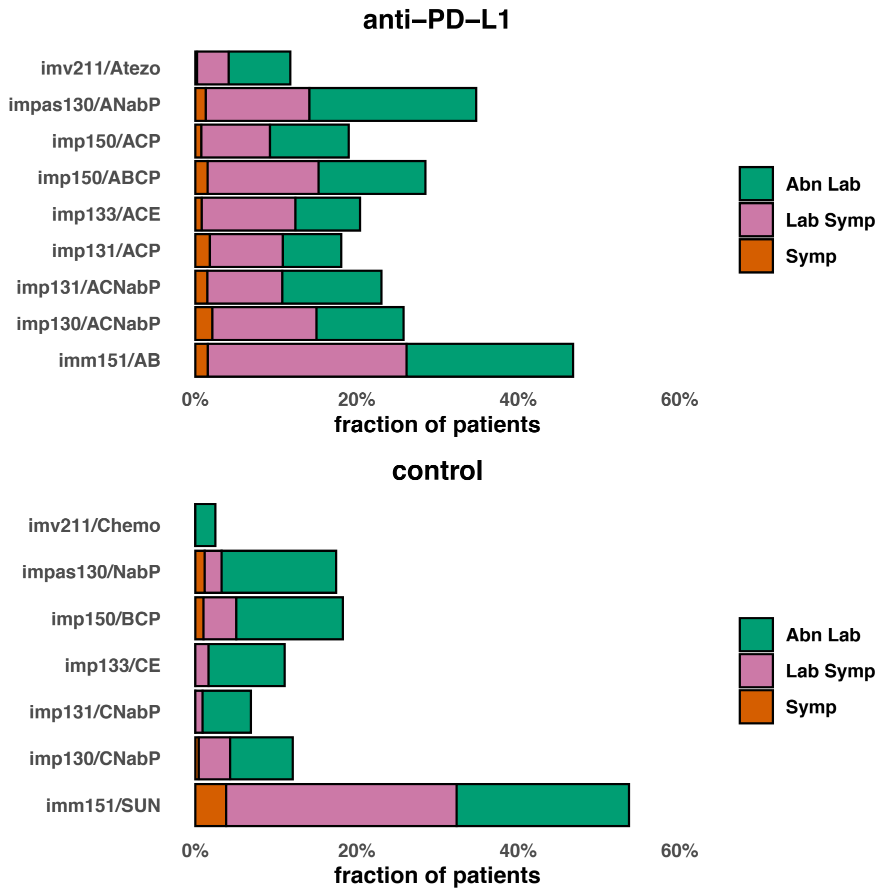

Supplementary Figure 3. Fraction of patients with abnormal TSH measurements (TSH >5 mU/L) (Abn Lab), symptomatic hypothyroidism irAE (Symp), or symptomatic hypothyroidism with an abnormal TSH measurement within 7 days (Lab Symp). Trial and treatment combination abbreviations are provided in Supplementary Table 2.

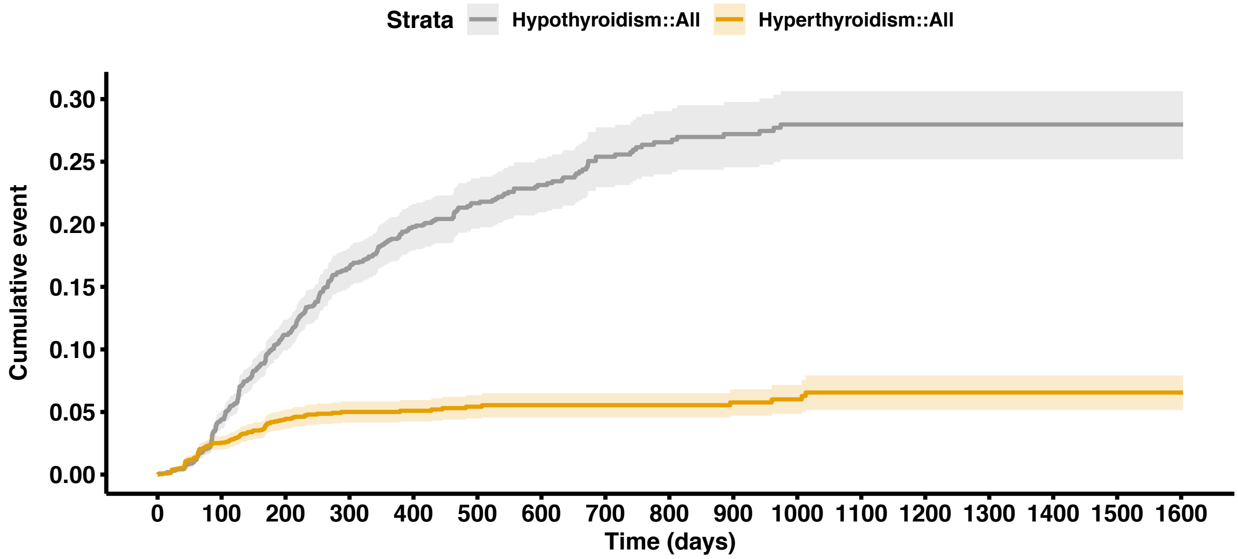

Number at risk

Hypothyroidism::All (grey)	3552	2648	1661	1096	808	646	529	428	352	308	259	200	140	93	40	11	1
Hyperthyroidism::All (orange)	3552	2697	1804	1261	975	800	668	561	469	418	353	270	187	121	53	15	1

Cumulative number of events

Hypothyroidism::All (grey)	0	135	296	383	419	436	447	461	467	470	473	473	473	473	473	473	473
Hyperthyroidism::All (orange)	0	82	127	136	137	140	141	141	141	142	143	145	145	145	145	145	145

Supplementary Figure 4. Cumulative event plot for hypothyroidism and hyperthyroidism in all safety evaluable anti-PD-L1 treated patients. Shaded areas designate 95% confidence intervals.

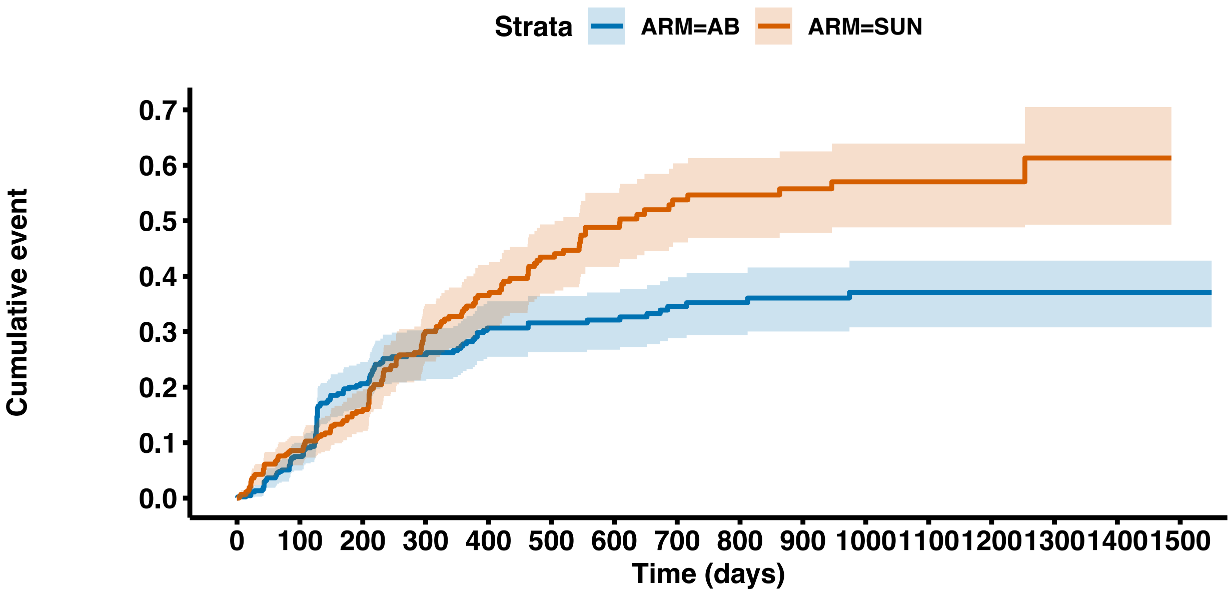

Number at risk

ARM=AB (blue)	451	360	251	199	165	141	124	100	77	66	62	53	39	23	10	2
ARM=SUN (orange)	445	350	244	166	127	94	69	52	47	38	30	28	15	5	2	0

Cumulative number of events

ARM=AB	0	32	80	96	108	110	111	115	116	117	118	118	118	118	118	118
ARM=SUN	0	37	61	98	112	125	133	139	140	141	142	142	142	143	143	143

Supplementary Figure 5. Cumulative event plot for hypothyroidism comparing the atezolizumab and bevacizumab combination arm and the sunitinib arm of the IMmotion151 trial in renal cell carcinoma. Shaded areas designate 95% confidence intervals. AB = Atezolizumab and bevacizumab, SUN = sunitinib.

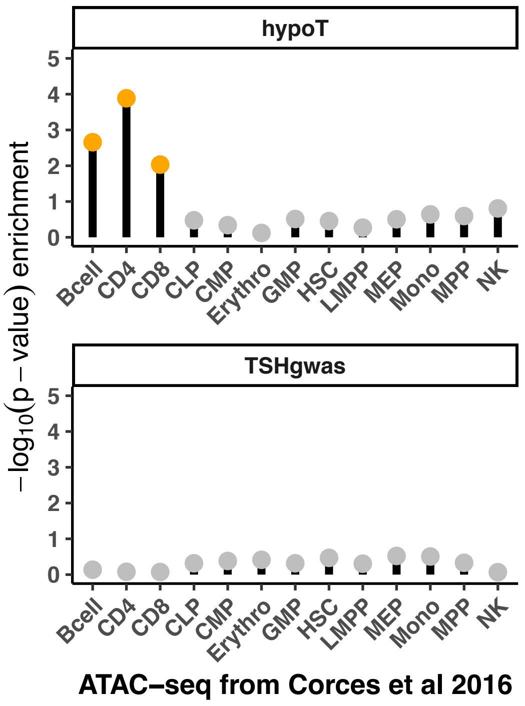

Supplementary Figure 6. -log10(p-values) for LD score regression heritability enrichment for the UKBB hypothyroidism GWAS and a GWAS of TSH levels in accessible chromatin measured by ATAC-seq across hematopoiesis. Orange circles designate enrichments significant at a false discovery rate (FDR) of 10% as estimated by the Benjamini-Hochberg method. Cell types are coded as follows: C=common; P=progenitor; M=myeloid; Erythro=Erythrocyte; granulocyte macrophage=GM; HSC = hematopoietic stem cell; LMP=lymphoid-primed multipotent; Mono=monocyte.

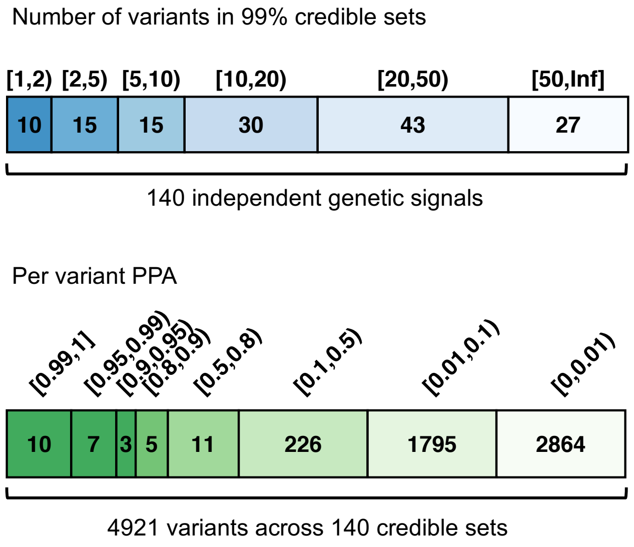

Supplementary Figure 7. Properties of the credible sets identified by fine mapping. Top, number of variants in each of 140 credible sets identified. Bottom, distribution of the posterior probability of association (PPA) of variants belonging to the credible sets.

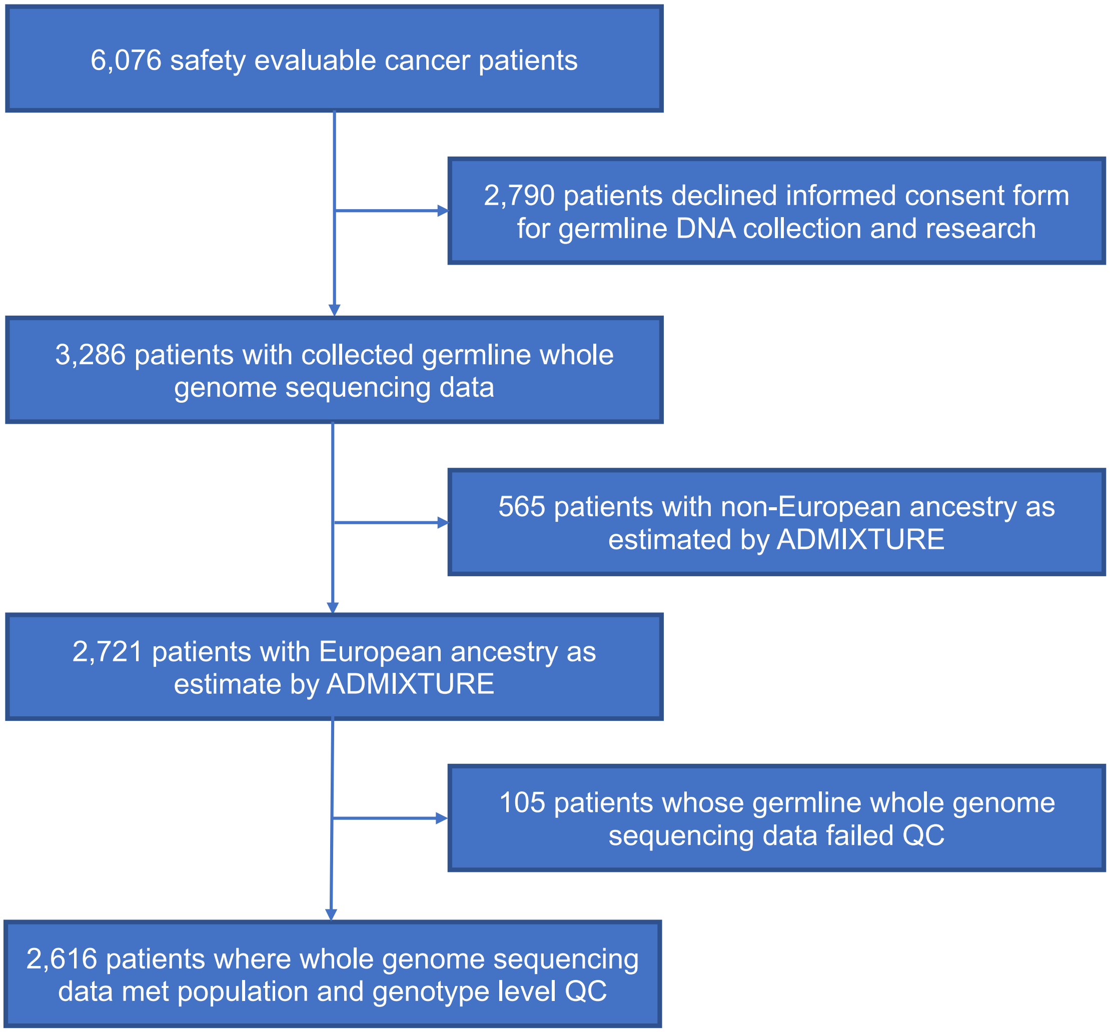

Supplementary Figure 8. Consort diagram illustrating the reasons for removal of patients and whole genome sequencing data on the basis of informed consent, European ancestry, and quality control (QC) filters. See also Supplementary Table 2.

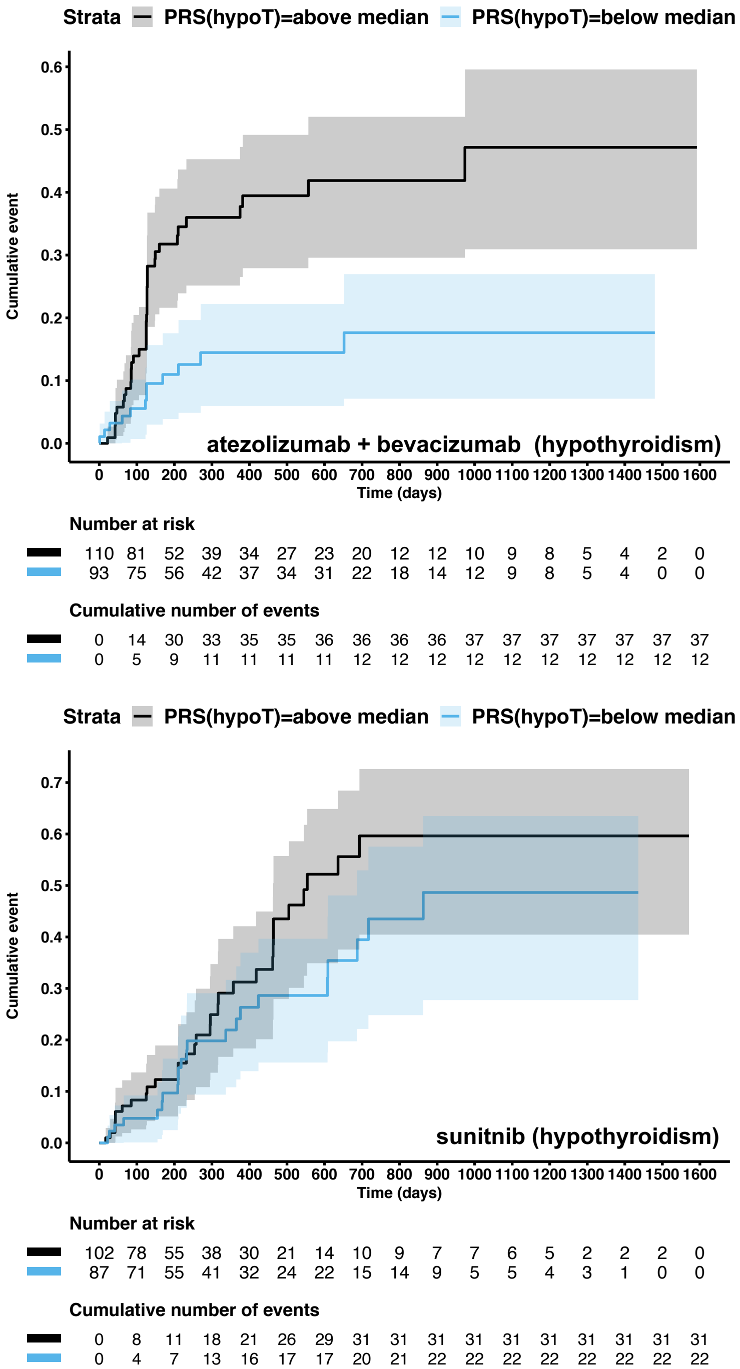

Supplementary Figure 9. Cumulative incidence plot comparing risk of hypothyroidism in renal cell carcinoma patients from the IMmotion151 trial treated with atezolizumab and bevacizumab as compared patients treated with sunitinib stratified by above and below median PRS for hypothyroidism. The median value was computed across all patients with genetic data including those in the control arms. Shaded regions designate the 95% confidence intervals.

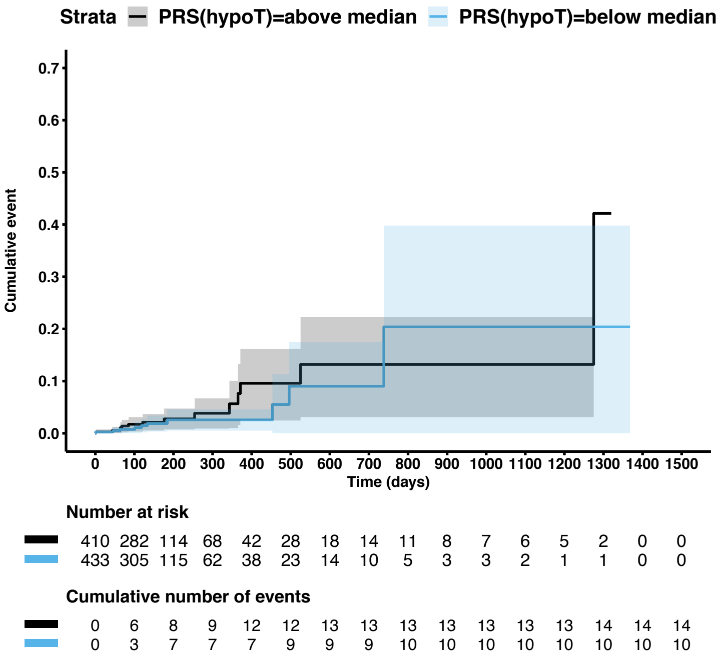

Supplementary Figure 10. Cumulative incidence plot for hypothyroidism in cancer patients in the control arms, excluding sunitinib treated patients, stratified by above and below median hypothyroidism PRS. The median value was computed across all patients with genetic data including those in the control arms. Shaded regions designate the 95% confidence intervals.

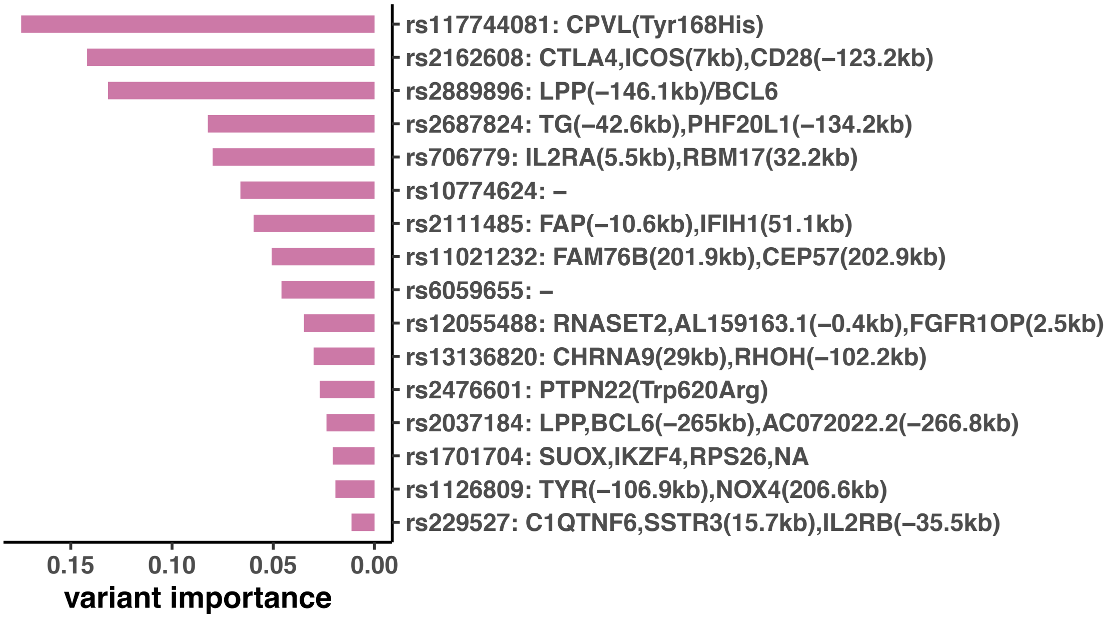

Supplementary Figure 11. Estimated importance of variants from the vitiligo PRS in a survival lasso model for time to hypothyroidism irAE in atezolizumab treated patients. The genes whose TSS are spanned by the credible set to which the lasso retained variant belongs are provided with no trailing parentheses. The two closest genes in genomic distance between credible set ends are indicated by trailing parenthesis containing distance in kilobases (kb). Only genes who have TSS within 500kb are reported. "-" designates credible sets that span more than 3 TSS.

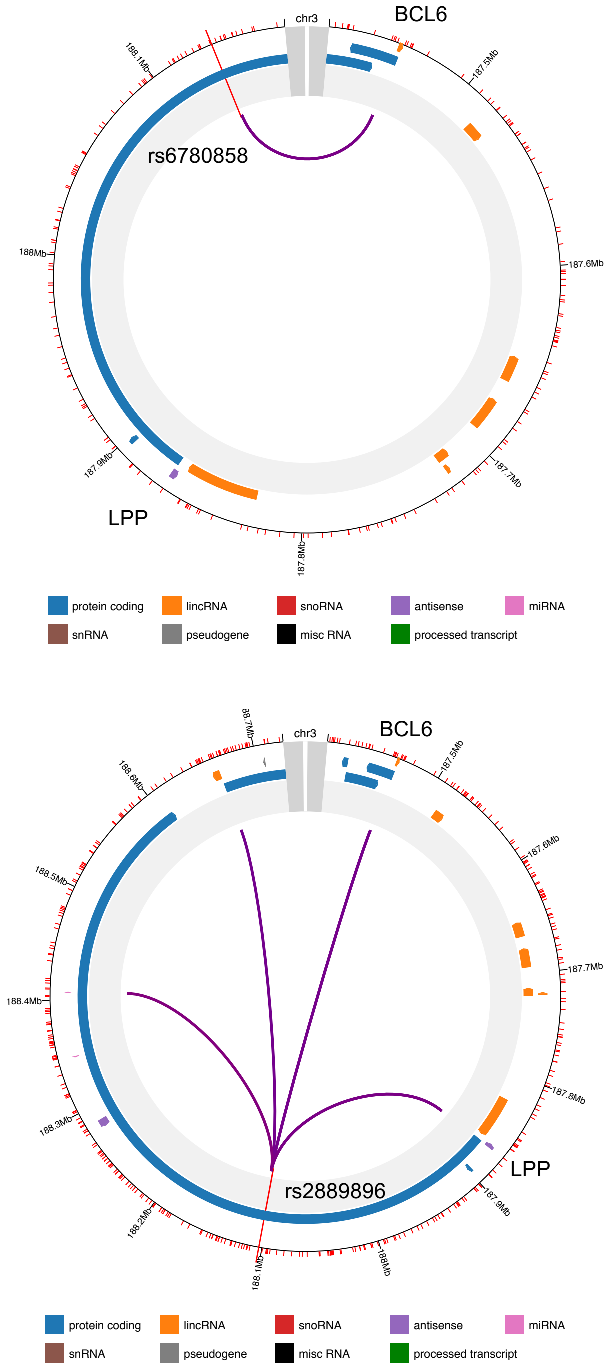

Supplementary Figure 12. Chord plot illustrating promoter capture Hi-C interactions between variants retained in the hypothyroidism and vitiligo PRSs within the LPP gene. Chord plot was generated using the Capture Hi-C Plotter (https://www.chicp.org) illustrating interactions with score ≥5 in the GM12878 lymphoblastoid cell line data set from Mifsud et al[15]. The transcription start sites of the LPP and BCL genes are highlighted. The position of the PRS variants in LPP are shown by the red line. Both rs6780858 ($p=4.2x10^{-12}$) and rs2889896 ($p=4.4x10^{-12}$) were eQTLs in blood for BCL6 in eQTLGen (https://www.eqtlgen.org/).

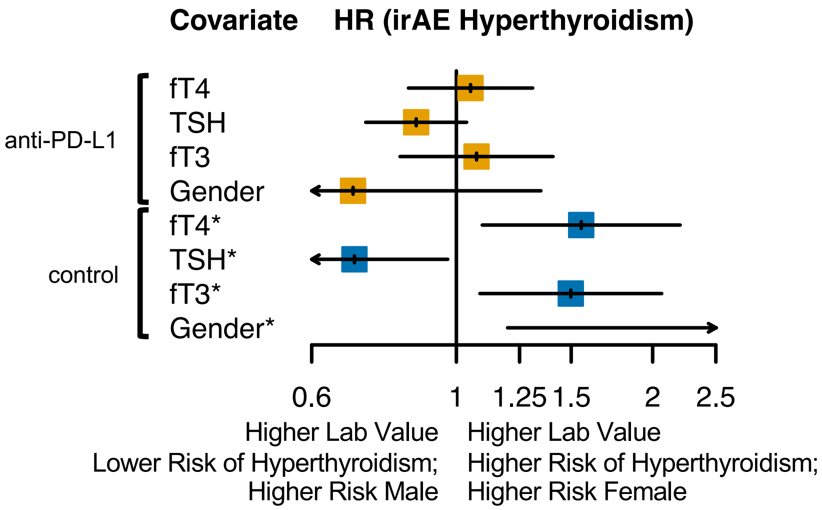

Supplementary Figure 13. Individual participant data meta-analysis assessing the association between hyperthyroidism irAE and potential pre-treatment risk factors in a multivariable mixed effects Cox model fit to data from (N=3,234) atezolizumab and (N=2,297) standard of care treated cancer patients in the safety evaluable population with pre-treatment thyroid hormone measurements across the 7 clinical trials analyzed. Measurements were normalized across patients by normalization to the quantiles of a standard normal distribution and modelled as random effects. Point estimates and 95% CI for HR for hypothyroidism expressed in unit normalized hormone levels after fitting the model. TSH = pre-treatment measured thyroid stimulating hormone; fT4 = free thyroxine; fT3 = free triiodothyronine. Gender is encoded as 1=female and 0=male. p-values for a two-sided Wald test that the log-HR is non-zero for fT4 p=0.65, TSH p=0.11, and gender p=0.28 in atezolizumab treated patients and fT4 p=0.013, TSH p=0.032, and gender p=0.019 in standard of care treated patients. Meta-analysis: *p < 0.05, **p < 0.01, ***p < 0.001, ****p < 0.0001

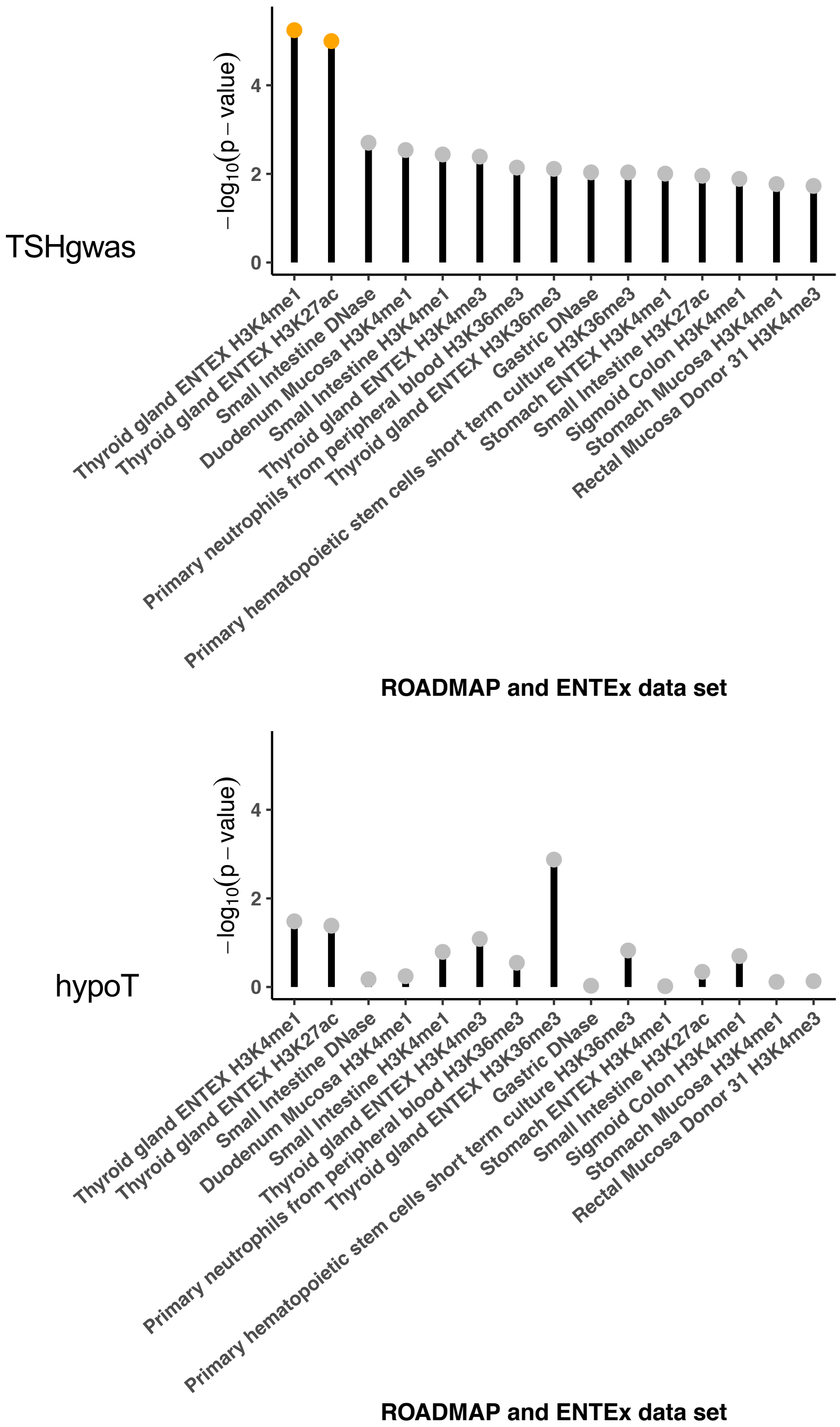

Supplementary Figure 14. Stratified LD score regression enrichment over the baseline model for ROADMAP Epigenomic and ENTex annotations sorted the 15 smallest enrichment p-values (out of 489 annotations tested) for the TSH GWAS. Bottom, corresponding enrichment p-values for the hypothyroidism GWAS from UKBB for the same annotations as above. Orange circles designate enrichments significant at an FDR of 10%, estimated using the Benjamini-Hochberg method.

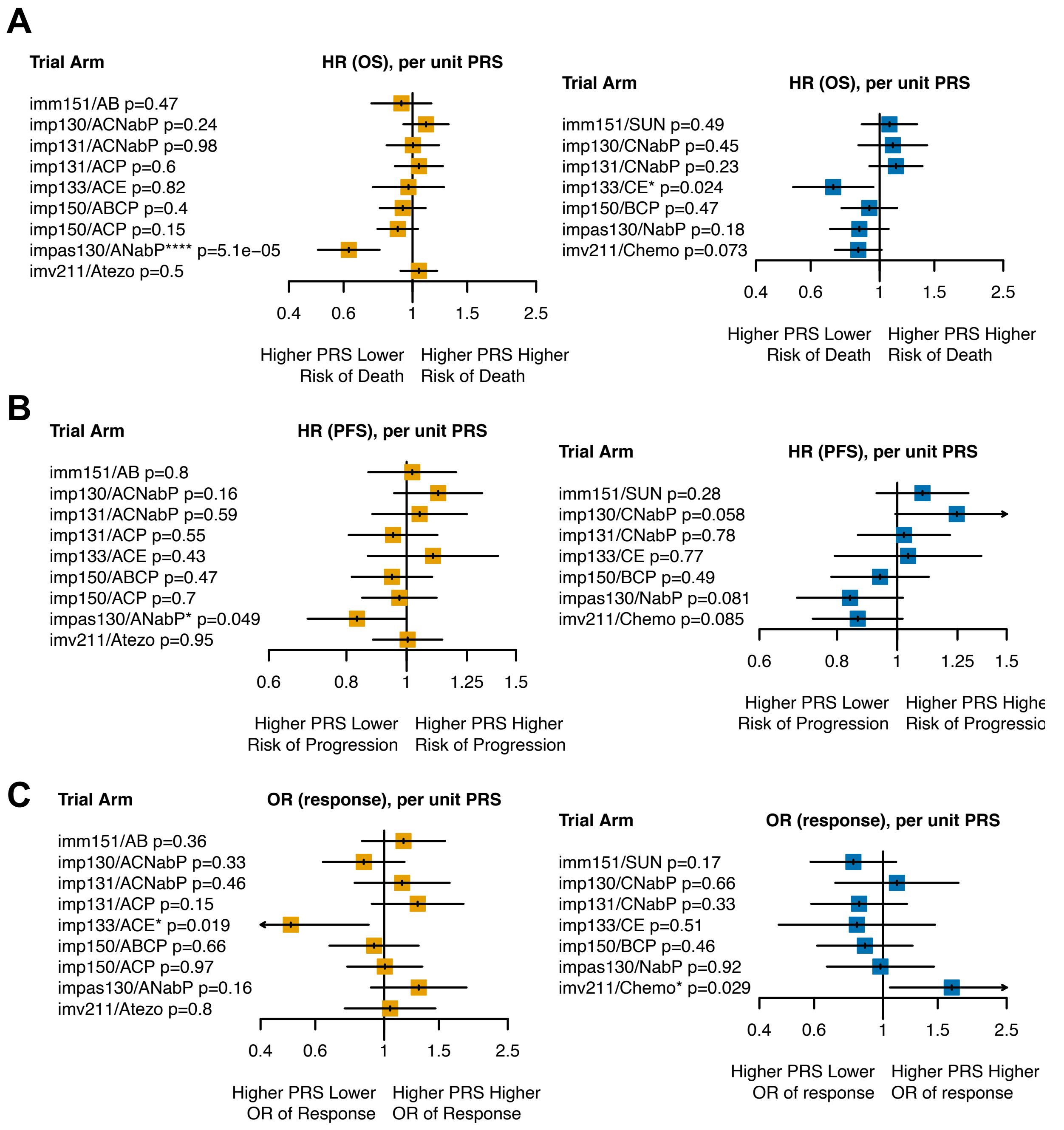

Supplementary Figure 15. Association between the hypothyroidism PRS and the following outcomes in arms of the atezolizumab trials analyzed in this study: (A) overall survival (OS); (B) progression free survival (PFS); (C) best confirmed objective response by RECIST (0 = progressive disease or stable disease versus 1 = partial response or complete response). Estimates of the hazard ratios (HRs) are expressed in per unit PRS which was normalized by quantile normalization to a standard normal distribution. Odds ratios (ORs) are also expressed in per unit normalized PRS. Lines designate 95% confidence intervals and center ticks designate estimates corresponding to the PRS coefficient in a Cox model (for PFS and OS) and OR (for response) adjusted for genotype eigenvectors, baseline ECOG status, and presence of pre-treatment liver metastases by inclusion of these as covariates in the models. p-values are provided for a two-sided Wald test that the log-HRs or log-ORs are significantly non-zero for the PRS coefficient in the Cox and logistic regression models respectively. Analyses was limited to European (EUR) ancestry individuals in each trial arm. Sample sizes per trial arm for EUR ancestry patients are provided in Supplementary Table 2. *p < 0.05 (not significant after accounting for multiple testing) ****p < 0.0001 (p-value significant after accounting multiple testing by Bonferroni correction)

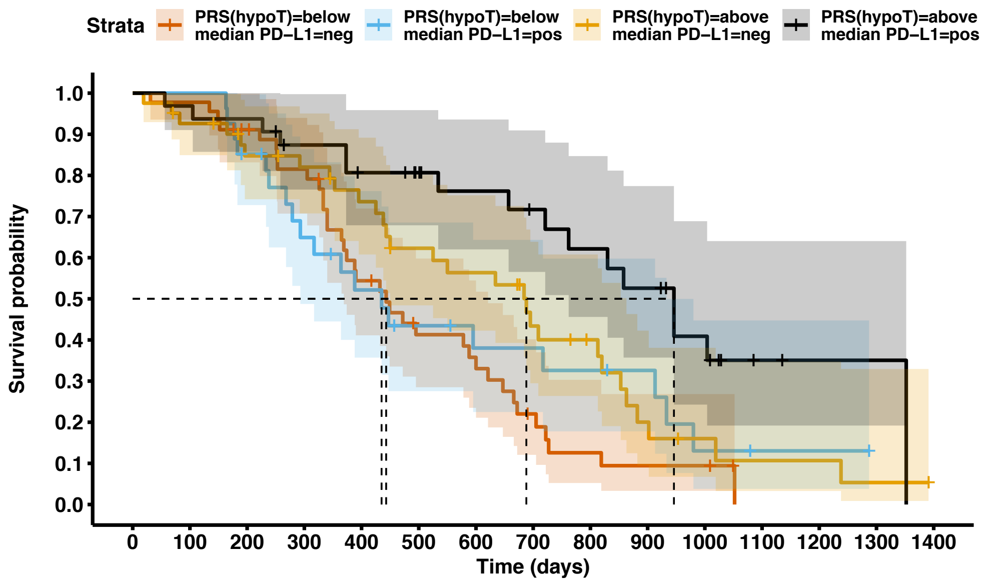

Number at risk

	45	44	39	34	22	15	13	7	4	3	3	0	0	0	0
	27	27	22	16	12	9	7	7	6	5	2	1	1	0	0
	41	37	32	30	26	21	19	13	10	5	3	2	2	1	0
	32	31	30	26	23	20	17	15	13	11	7	2	1	1	0

Supplementary Figure 16. Kaplan-Meier plot of overall survival of TNBC patients from the atezolizumab plus nab-paclitaxel arm of IMpassion130. Patients were stratified on tumor PD-L1 positivity and by high (above median) and low (below median) hypothyroidism PRS. Dashed lines show censoring events and shaded regions provide 95% confidence intervals. Vertical and horizontal lines designate the median survival time for each group. Abbreviations: pos = positive, neg = negative.

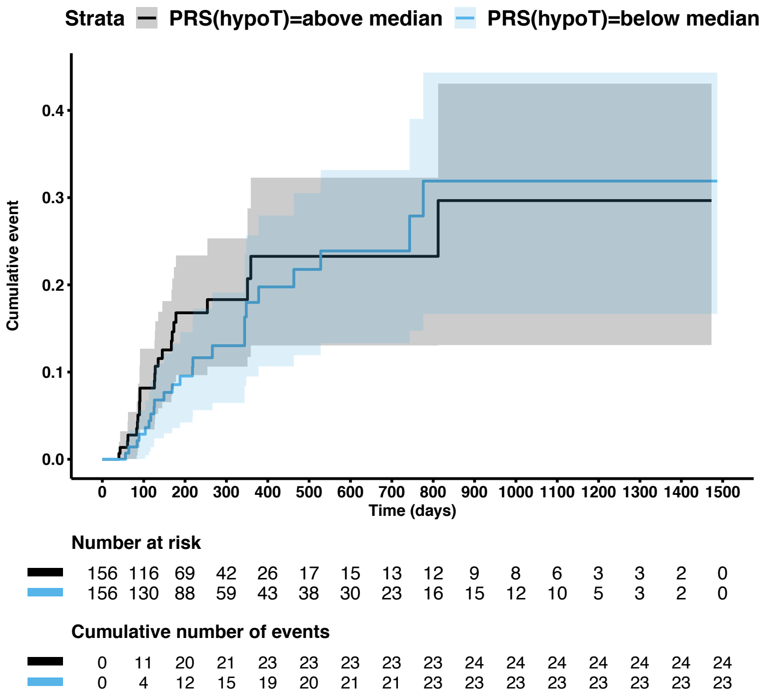

Supplementary Figure 17. Cumulative incidence plot for time to occurrence of hypothyroidism irAE in atezolizumab treated cancer patients that did not meet our cutoff for European ancestry (EUR < 0.7). Patients were split by above or below median hypothyroidism PRS values. Shaded regions designate the 95% confidence intervals.

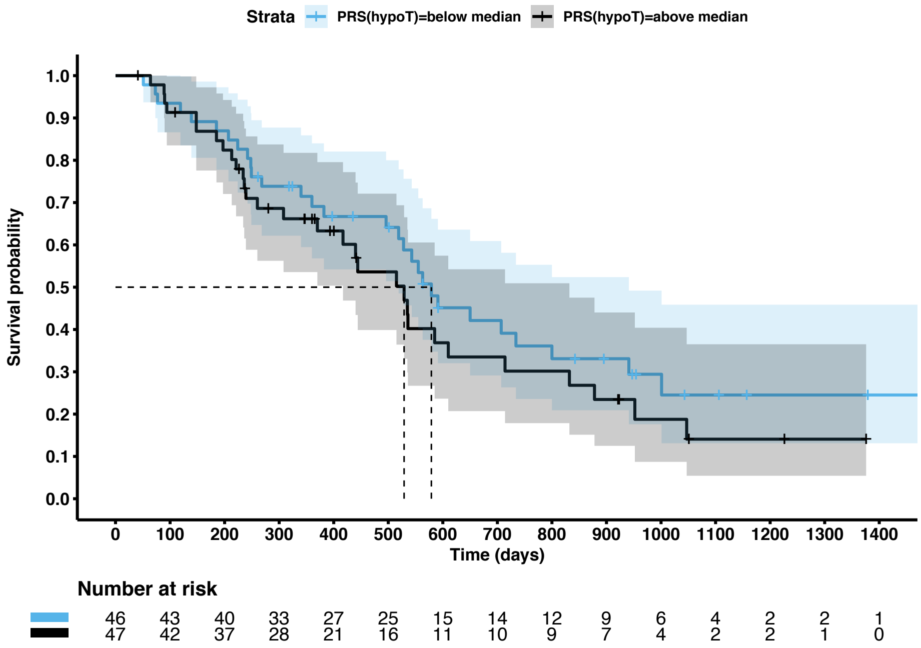

Supplementary Figure 18. Kaplan-Meier plot of overall survival from TNBC patients from the atezolizumab plus nab-paclitaxel arm of IMpassion130 not meeting our cutoff for European ancestry (EUR < 0.7) split into two groups by above and below median hypothyroidism PRS. Shaded regions designate 95% confidence intervals.

References

1. Powles, T. *et al.* Atezolizumab versus chemotherapy in patients with platinum-treated locally advanced or metastatic urothelial carcinoma (IMvigor211): a multicentre, open-label, phase 3 randomised controlled trial. *Lancet* **391,** 748–757 (2018).
2. Socinski, M. A. *et al.* Atezolizumab for First-Line Treatment of Metastatic Nonsquamous NSCLC. *N. Engl. J. Med.* **378,** 2288–2301 (2018).
3. Jotte, R. *et al.* Atezolizumab in Combination With Carboplatin and Nab-Paclitaxel in Advanced Squamous NSCLC (IMpower131): Results From a Randomized Phase III Trial. *J. Thorac. Oncol.* **15,** 1351–1360 (2020).
4. West, H. *et al.* Atezolizumab in combination with carboplatin plus nab-paclitaxel chemotherapy compared with chemotherapy alone as first-line treatment for metastatic non-squamous non-small-cell lung cancer (IMpower130): a multicentre, randomised, open-label, phase 3 trial. *Lancet Oncol.* **20,** 924–937 (2019).
5. Horn, L. *et al.* First-Line Atezolizumab plus Chemotherapy in Extensive-Stage Small-Cell Lung Cancer. *N. Engl. J. Med.* **379,** 2220–2229 (2018).
6. Rini, B. I. *et al.* Atezolizumab plus bevacizumab versus sunitinib in patients with previously untreated metastatic renal cell carcinoma (IMmotion151): a multicentre, open-label, phase 3, randomised controlled trial. *Lancet* **393,** 2404–2415 (2019).
7. Schmid, P. *et al.* Atezolizumab and Nab-Paclitaxel in Advanced Triple-Negative Breast Cancer. *N. Engl. J. Med.* **379,** 2108–2121 (2018).
8. Schmid, P. *et al.* Atezolizumab plus nab-paclitaxel as first-line treatment for unresectable, locally advanced or metastatic triple-negative breast cancer (IMpassion130): updated efficacy results from a randomised, double-blind, placebo-controlled, phase 3 trial. *Lancet Oncol.* **21,** 44–59 (2020).
9. Teumer, A. *et al.* Genome-wide analyses identify a role for SLC17A4 and AADAT in thyroid hormone regulation. *Nat. Commun.* **9,** 4455 (2018).
10. Bycroft, C. *et al.* The UK Biobank resource with deep phenotyping and genomic data. *Nature* **562,** 203–209 (2018).
11. Censin, J. C. *et al.* Childhood adiposity and risk of type 1 diabetes: A Mendelian randomization study. *PLoS Med.* **14,** e1002362 (2017).
12. Jin, Y. *et al.* Genome-wide association studies of autoimmune vitiligo identify 23 new risk loci and highlight key pathways and regulatory variants. *Nat. Genet.* **48,** 1418–1424 (2016).
13. Zhou, W. *et al.* Efficiently controlling for case-control imbalance and sample relatedness in large-scale genetic association studies. *Nat. Genet.* **50,** 1335–1341 (2018).
14. de Bakker, P. I. W. *et al.* Practical aspects of imputation-driven meta-analysis of genome-wide association studies. *Hum. Mol. Genet.* **17,** R122-8 (2008).
15. Mifsud, B. *et al.* Mapping long-range promoter contacts in human cells with high-resolution capture Hi-C. *Nat. Genet.* **47,** 598–606 (2015).

Supplementary Note 1

Ethics Committees (EC) and Institutional Review Boards (IRB) at each study site for each clinical trial approved the clinical trial protocol, the main study ICF, and the RBR ICF. The EC and IRB for each clinical trial and study site are provided below:

IMvigor211
1. NRES Committee London - West London and GTAC, UNITED KINGDOM
2. Asan Medical Center Ethics Committee, Seoul, KOREA, REPUBLIC OF
3. Samsung Medical Center EC, Seoul, KOREA, REPUBLIC OF
4. Seoul National University Hospital, KOREA, REPUBLIC OF

5. Austin Health HREC, Research Ethics Unit, AUSTRALIA
6. COMITATO ETICO UNICO REGIONALE VIA POZZUOLO 330, UDINE
7. COMITATO ETICO DELLA FONDAZIONE IRCCS "ISTITUTO NAZIONALE DEI TUMORI"-
8. VIA G. VENEZIAN 1, MILANO
9. COMITATO ETICO CARDARELLI-SANTOBONO VIA A. CARDARELLI, 9 NAPOLI
10. COMITATO ETICO IRCCS DI CANDIOLO STRADA PROVINCIALE 142 CANDIOLO (TO)
11. Comitato Etico Provinciale Modena
12. Comitato Etico Di Area Vasta Romagna E Irst, Via Piero Maroncelli 40, 47014, Meldola, Emilia-Romagna, ITALY
13. COMITATO ETICO LAZIO 1 CIRCONVALLAZIONE GIANICOLENSE 87 – ROMA
14. COMITATO ETICO REGIONE TOSCANA - AREA VASTA SUD EST VIALE BRACCI 16 - SIENA
15. COMITATO ETICO DELLA PROVINCIA DI BERGAMO
16. Kantonale Ethikkommission Zürich (KEK)
17. Ege Üniversitesi Klinik Araştırmalar Etik Kurulu
18. Ethikkommission der Stadt Wien gemäß KAG, AMG und MPG
19. Hospital Ramón y Cajal ;Comité Ético de Investigación Clínica Ctra. Colmenar Viejo, km 9,1 28034 MADRID
20. UZ Gent, Commissie voor Medische Ethiek, C. Huysmanslaan 10, 9000 Gent – BELGIUM
21. Varsinais-Suomen shp Eettinen toimikunta
22. National Ethics Committee, Ministry of Health and Social Wellfare, 284, Messogion Avenue, 15562, Cholargos, GREECE
23. UBC BCCA Research Ethics Board (BCCA REB), 902-750 West Broadway, Fairmont Medical Building, V5Z 1H8, Vancouver, British Columbia, CANADA
24. CPP Sud Ouest Et Outre Mer IV, Hôpital Jean Rebeyrol, avenue du Buisson, 87042, Limoges, FRANCE
25. Ontario Cancer Research Ethics Board, MaRS Centre, South Tower, 101 College Street, Suite 800, M5G 1L7, Toronto, Ontario, CANADA
26. McGill University; McGill University; Ethics Board, 3655 Promenade Sir William Osler - 6th Floor, H3G 1Y6, Montreal, Quebec, CANADA
27. Lakeridge Health Research Ethics Board, 1 HOSPITAL COURT, L1G 2B9, OSHAWA, Ontario, CANADA
28. HREBA - Health Research Ethics Board of Alberta - Cancer Committee, c/o Alberta Innovates - Health Solutions, Suite 1500 - 10104, 103 Avenue NW, T5J 4A7, Edmonton, Alberta, CANADA
29. Regionala Etikprövningsnämnden i Stockholm, Box 289, Karolinska Institute, Nobelsväg 12A, 171 77, Stockholm, SWEDEN
30. COMITATO ETICO AREA CREMONA MANTOVA E LODI, Viale Concordia 1, Servizio di Farmacia, 26100, Cremona, Lombardia, ITALY
31. COMITATO ETICO REGIONE TOSCANA - AREA VASTA CENTRO LARGO BRAMBILLA, 3 FIRENZE
32. SEZ DEL CE IRCCS IST TUMORI G PAOLO II BA C/O FONDAZIONE CASA SOLLIEVO DELLA SOFFERENZA SG ROTONDO - VIALE CAPPUCCINI - SAN GIOVANNI ROTONDO (FG)
33. Research Ethics Committee, Nat. Taiwan Univ. Hosp., No.1, Changde Street, Zhongzheng District, 100, TAIPEI, TAIWAN
34. TVGH Institutional Review Board, No.201, Shih-Pai Road, Sec.2, 112, Taipei, TAIWAN
35. MEC-U | Medical Research Ethics Committees United
36. EC of FSBI Privolzhsky Federal Medical Research Centre, 18-1 Vergne Volzhskaya embankment, 603155, Nizhny Novgorod, RUSSIAN FEDERATION
37. CEIC - Comissão de Ética para Investigação Clínica, CEIC - Comissão de Ética para Investigação Clínica, Av. do Brasil, 53 - Pav 17-A ,Parque da Saúde de Lisboa, 1749-004, Lisboa, PORTUGAL
38. Niezależna Komisja Bioetyczna ds Badań Naukowych, Dębinki 7, budynek nr 1, III piętro, 80-211, Gdańsk, POLAND
39. Comisia Națională de Bioetică a Medicamentului și a Dispozitivelor Medicale- Sos, Sos. Stefan cel Mare nr. 19-21, Pavilion K, sector 2, 020125, București, ROMANIA
40. The IRB, Taichung Veterans General Hospital, No. 1650, Taiwan Boulevard, Sect. 4, 407, Taichung, TAIWAN
41. E.C. of Altai Oncological Center, Nikitina street, 77, 656049, Barnaul, RUSSIAN FEDERATION

42. Medical Research Council, Ethics Committee for Clinical Pharmacology, Arany J. u. 6-8., 1051, Budapest, HUNGARY
43. Etická Komise Fakultni Nemocnice Olomouc, I.P. PAVLOVA 6, 775 20, OLOMOUC, CZECH REPUBLIC
44. Eticka komise Masarykova onkologicke o ustavu, Zluty Kopec 7, 656 53, Brno, CZECH REPUBLIC
45. Komisija Republike Slovenije za Medicinsko Etiko, Ministrstvo za zdravje, Stefanova 5, 1000, Ljubljana, SLOVENIA
46. IRB, ChinaMedicalUniversityHospital, No. 2 Yuh Der Road, 404, Taichung, TAIWAN
47. CPP Sud-Ouest et Outre-mer IV- (Docteur Claire Demiot)Centre hospitalier Esquirol Cabanis Haut- 15 rue du Docteur Marcland- 87025 LIMOGES Cedex
48. Ethikkommission Technische Universität München, Fakultät für Medizin Ismaninger Str. 22 81675 München
49. De Videnskabsetiske Komitéer for Region Hovedstaden, Regionsgården, Kongens Vænge 2, 3400 Hillerød; Denmark De Videnskabsetiske Komitéer for Region Hovedstaden, Regionsgården, Kongens Vænge 2, 3400 Hillerød, Denmark
50. REK Sør-Øst, P.b. 1130 Blindern, 0318 Oslo, NORWAY
51. Ethics Committee Institute for Oncology of Vojvodina, Put doktora Goldmana 4, 21204, Sremska Kamenica, SERBIA
52. Ethics Committee of Institute of Oncology & Radiology, PASTEROVA 14, 11000, BELGRADE, SERBIA
53. Commission centrale d'éthique, Hôpital Universitaire Genève
54. Kantonale Ethikkommission Bern KEK
55. Ethikkommission Ostschweiz (EKOS)
56. Duke University Health System; Institutional Review Board for Clinical Investigations
57. WESTERN INTERNATIONAL REVIEW BOARD
58. Vanderbilt University Institutional Review Board
59. Western Institutional Review Board
60. Lehigh Valley Health Network IRB
61. MedStar Health Research Institute-Georgetown Univ. Oncology IRB
62. Iwate Medical University Institutional Review Board, 19-1, Uchimaru, Morioka-shi, 020-8505, Iwate, JAPAN
63. Hokkaido University Hospital Institutional Review Board, Kita14-jo,Nishi5-chome,Kita-ku,Sapporo, 060-8648, Hokkaido, JAPAN
64. Institutional Review Board of Osaka International Cancer Institute, 3-3 Nakamichi 1-Chome, Higashinari-ku, 537-8511, Osaka, JAPAN
65. SHIKOKU CANCER CENTER INSTITUTIONAL REVIEW BOARD, 160 Minamiumemoto-Machi-Kou; Matsuyama-Shi, 791-0280, Ehime, JAPAN
66. University of Tsukuba Hospital Institutional Review Board, 2-1-1 Amakubo, Tsukuba-shi, 305-8576, Ibaraki, JAPAN
67. Gunma University Hospital Institutional Review Board, 3-39-15 Showa-machi,Maebashi-shi, 371-8511, Gunma, JAPAN
68. The Cancer Institute Hospital of JFCR Institutional Review Board, 3-8-31 Ariake Koto-Ku, 135-8550, Tokyo, JAPAN
69. Osaka University Hospital Institutional Review Board, 2-15, Yamadaoka, Suita-shi, 565-0871, Osaka, JAPAN
70. Yokohama City University Hospital Institutional Review Board, 3-9 Fukuura,Kanazawa-ku,Yokohama-shi, 236-0004, Kanagawa, JAPAN
71. Kindai University Hospital Institutional Review Board, 377-Ohnohigashi, Osaka-Sayama-shi, 589-8511, Osaka, JAPAN
72. Chiba Cancer Center Institutional Review Board, 666-2 Nitona-cho Chuo-ku, Chiba-shi, 260-8717, Chiba, JAPAN
73. Toranomon Hospital and Toranomon Hospital Kajigaya Institutional Review Board, 2-2-2 Toranomon, Minato-ku, 105-8470, Tokyo, JAPAN
74. Tokushima University Hospital Institutional Review Board
75. Shizuoka Cancer Center Ethical Review Board for Business Clinical Studies, 1007 Shimonagakubo Nagaizumi-cho, Suntou-gun, 411-8777, Shizuoka, JAPAN

76. The Institutional Review Board of Kumamoto University Hospital, 1-1-1 Honjo, Chuo-ku, Kumamoto-shi, 860-8556, Kumamoto, JAPAN
77. National Cancer Center Institutional Review Board, 5-1-1 Tsukiji Chuo-Ku, 104-0045, Tokyo, JAPAN
78. Niigata Cancer Center Hospital Institutional Review Board, 2-15-3 Kawagishi-cho, Chuo-ku, Niigata-shi, 951-8566, Niigata, JAPAN
79. Nagoya university Hospital IRB, 65 tsurumai-cho, showa-ku, nagoya-shi, 466-8560, Aichi, JAPAN
80. Nippon Medical School Hospital Institutional Review Board, 1-1-5 Sendagi, Bunkyo-ku, 113-8603, Tokyo, JAPAN
81. NATIONAL CANCER CENTER EAST INST. REVIEW BOARD, 6-5-1 KASHIWANOHA, KASHIWA, 277-8577, CHIBA, JAPAN
82. Institutional Review Board of Hirosaki University School of Medicine and Hospital, 53 Honcho, Hirosaki-shi, 036-8563, Aomori, JAPAN
83. Harasanshin Hospital Institutional Review Board, 1-8 Taihaku-machi, Hakata-ku, Fukuoka-shi, 812-0033, Fukuoka, JAPAN
84. CPP Sud Ouest Et Outre Mer IV
85. EC of State Institution of Healthcare Stavropol regional clinical oncology dispensary, 182a, Octyabrskaya Str., 355047, Stavropol, RUSSIAN FEDERATION
86. CEIC Hospital Clínico Universitario de Valencia, Avda. Menendez Pelayo 4, accesorio, 46010, Valencia, VALENCIA, SPAIN
87. Comité Ético de Investigación Clínica Agencia de Ensayos-HOSPITAL RAMÓN Y CAJAL, CTRA. DE COLMENAR VIEJO, KM 9,1, 28034, MADRID, MADRID, SPAIN
88. CEIC Hospital General Universitario de Valencia, Avda. Tres Cruces s/n, 46014, Valencia, VALENCIA, SPAIN Comité Ético de Investigación Clínica Agencia de Ensayos- HOSPITAL RAMÓN Y CAJAL, CTRA. DE COLMENAR VIEJO, KM 9,1, 28034, MADRID, MADRID, SPAIN
89. CEIC Hospital Clínico Universitario Virgen de la Victoria, Campus de Teatinos s/n, Garantía de Calidad, 29010, Málaga, MÁLAGA, SPAIN

IMpower150

1. Cernicus Group Independent Review Board; 1 Triangle Drive Suite 100 PO Box 110605 Research Triangle Park North Carolina 27709 United States
2. Western Institutional Review Board; 1019 39th Avenue Southeast Suite 120 Puyallup Washington 98374 United States
3. Melbourne Health Human Research Ethics Committee
4. Tasmania Health and Medical Human Research Ethics Committee
5. Asociacion Benefica Prisma
6. Comité institucional de ética en investigación Instituto Nacional de Enfermedades Neoplásicas
7. Singhealth Centralised Institutional Review Board
8. Institutional Review Chung-Ho Board Memorial Kaohsiung Hospital Medical University
9. Institutional Review Board Taipei Veterans General Hospital
10. Institutional Review Board, Changhua Christian Hospital
11. Institutional Review Board of the Chi Mei Medical Center
12. The Institutional Review General Board Hospital of Taichung Veterans
13. Institutional Review Board of Tri-Service General Hospital
14. Cheng-Hsin General Hospital Institutional Review Board
15. Consejo de Evaluación Ética de investigación en Salud -CoEIS
16. Ministerio de Salud - Provincia de Rio Negro
17. Comité independiente de Ética en investigación clínica "Dr. Carlos A. Barclay”
18. Consejo de Evaluación Ética de investigación en Salud – CoEIS
19. Comité de ética en investigaci6n Fundación Oncosalud Comisión Conjunta de investigación en Salud (CCIS)
20. Comite de Etica Centro de Oncología e investigación Buenos Aires
21. Comisión Conjunta de investigación en Salud (CCIS)
22. Comité de Ética independiente - Fundacion Sanatorio
23. Institutional Review Board of Chang Gung Medical Foundation
24. Ethikkommission tor das Bundesland Salzburg; Sebastian-Stief-Gasse 2 Salzburg Salzburg 5010 Austria

25. The Ethics Committee for Clinical Trials of Medicinal Products
26. Chesapeake Institutional Review Board; 6940 Columbia Gateway Drive Suite 110 Columbia Maryland 21046 United States
27. St. Luke's Hospital & Health Network IRB; 801 Ostrum Street East Wing 4 Bethlehem Pennsylvania 18015 United States
28. St Charles Medical Center; 2500 Northeast Neff Road Bend Oregon 97701 United States
29. Saint Luke's Hospital Institutional Review Board; 915 East First Street Duluth Minnesota 55805 United States
30. Kantonale Ethikkommission Bern (KEK)
31. Comité de Ética Metropolitano Cientifico del Norte Servicio de Salud
32. Comitato Etico Area Vasta Nord Ovest presso Azienda Ospedaliero Universitaria Pisana di Pisa
33. Comitato Etico Cardarelli-Santobono
34. Comitato Etico Area Vasta Nord Ovest presso Azienda Ospedaliero Universitaria Pisana di Pisa
35. Comitato Etico San Martino 1ST 2
36. Comitato Etico Lazio 1
37. COMITATO ETICO DELL'UNIVERSITÀ' CAMPUS BIO- MEDICO DI ROMA
38. Medical Research Ethics Committees United
39. CPP Sud-Méditerranée 2; 270 Boulevard Sainte Marguerite Hôpital Sainte Margeurite Pavillon 9 Cedex 9 Marseille Bouches-du-Rhône 1327 4 France
40. Sir Charles Gairdner Hospital HREC
41. Bellberry Human Research Ethics Committee
42. Cabrini Human Research Ethics Committee
43. Comitato Etico Catania 1 presso A.O. Universitaria Policlinico Vittorio Emanuele di Catania
44. Comissão de Ética para a investigação Clínica - CEIC
45. Ethikkommission an der Universität Regensburg; Landshuter Straße 4 Raum 134, 1.0G. Regensburg 93047 Germany
46. Lakeridge Health REB; 1 Hospital Court Oshawa Ontario L 1 G 289 Canada
47. St. Joseh Mercy Health System Institutional Review Board #2 - Oncology Central IRB; 5301 East Huron River Drive Clinical Research Department, RHB 6017 Ann Arbor Michigan 48106 United States
48. Mercy Saint Vincent Medical Center Institutional Review Board; 2213 Cherrv Street Toledo Ohio 43608 United States
49. US Oncology Inc. Institutional Review Board; 10101 Woodloch Forest The Woodlands Texas 77380 United States
50. CEIC de la Corporación Sanitaria del Parc Taulí
51. CEIC de Cantabria
52. CEIC de Andalucía (CCEIBA)
53. CEIC Hospital Universitario La Paz
54. CEIC Fundaci6n Jimenez Diaz
55. CEIC Hospital Clínico Universitario de Valencia
56. CEIC Hospital General Universitario Gregorio Marañón
57. CEIC Hospital Clinico San Carlos
58. CEIC Grupo Hospital de Madrid
59. CEIC Pare de Salut Mar
60. CEIC Islas Baleares (CEIC-IB)
61. CEIC de Galicia (CAEI)
62. CEIC Hospital Clínic de Barcelona
63. Kaiser Permanente Southern California Institutional Review Board; 393 East Walnut Street, 2nd Floor Pasadena California 91188 United States
64. Research Ethics Committee of National Taiwan University Hospital
65. Institutional Review Board of Chung Shan Medical University Hospital
66. Ethikkommission für das Bundesland Salzburg; Sebastian-Stief-Gasse 2 Salzburg Salzburg 5010 Austria
67. CHU de Liège - Comité d'Éthique
68. The Ethics Committee for Products Clinical Trials of Medicinal Product
69. CEQ of Ml Dnipropetrovsk Kryvyi Rih Oncology Regional Council Dispensary of

70. CEQ of Treatment and Prevention Institution Volyn Regional Oncology Dispensary
71. CEQ of SI Institute of Medical NAMS Radiology n.a. S.P. Hryhoriw
72. CEQ of MI of Dnipropetrovsk Dnipropetrovsk City Regional Multifield Council Clinical Hospital #4 CEQ of Poltava Regional Clinical Oncology Dispensary of Poltava Regional Council
73. Commission on Ethics Clinical Questions Hospital of Uzhgorod Central City
74. Commission of Ethics Questions on the basis of the Chernivtsi Regional Clinical Oncology Dispensary
75. CEQ of MI of Zaporizhzhya Regional Council Zaporizhzhya Regional Clinical Oncology Dispensary
76. CEQ of Transcarpathian Regional Clinical Oncology Dispensary
77. CEQ of Municipal Noncommercial Institution Regional Center of Oncology
78. CEQ of Regional Municipal Institution Sumy Regional Clinical Oncology Dispensary
79. Comitato Etico Azienda Ospedaliera Universitaria Maggiore della Carita
80. Comitato Etico Policlinico Dell Universitario Universita Cattolica Agostino del Gemelli Sacra Cuore
81. Comitê de Ética em Pesquisa em Seres Humanos da Faculdade de Medicina de São José do Rio Prete
82. Comitê de Ética em Pesquisa em Seres Humanos do Hospital Secor
83. Comitê de Ética em Pesquisa em Seres Humanos da Universidade de Ribeirao Prete /UNAERP)
84. Comitê de Ética em Pesquisa em Seres Humanos da Irmandade da Santa Casa de Londrina
85. Comitê de Ética em Pesquisa em Seres Humanos da Liga Norte Riograndense Contra o Cancer
86. Comitê de Ética em Pesquisa Fundat,ao Pio XII Hospital de Câncer de Barretos
87. Comité de Ética en investigación de la Facultad de Medicina v Hospital Universitario
88. Missouri Baptist Medical Center Institutional Review Board; 3015 North Ballas Road St. Louis Missouri 63131 United States
89. University of California Irvine Institutional Review Board; 5171 California Avenue, Office Of Research Suite 150 Irvine California 92697 United States
90. University of California, San Diego Human Research Protections Program; 3350 La Jolla Village Drive San Diego California 92161 United States
91. Frederick Memorial Hospital Institutional Review Board; 400 West 7th Street Frederick Maryland 21701 United States
92. Mercy Medical Center IRB; 345 St. Paul Place Bunting Center, 7th Floor Baltimore Maryland 21202 United states
93. University of Chicago Hospitals Institutional Review Board; 5751 South Woodlawn Avenue McGiffert Hall Chicago Illinois 60637 United States
94. Mount Sinai Medical Center IRB; 4300 Alton Road Miami Beach Florida 33140 United States
95. Comité Provincial de Bioética - Ministerio de Salud de la Provincia de Santa Fe
96. Comité Independiente de Carlos Ética A. en Barclay investigación clinica "Dr.
97. Comité de Ética investigación de la Clínica Bajfo
98. Comité de Ética Clínica en Research Investigacion SA de CV
99. Comitê de Ética em Pesquisa da Universidade de Caxias do Sul
100. Comitê de Ética em Pesquisa rto Aleare do Hospital de Clínicas de
101. Comite de Etica em Pesquisa da Universidade Federal de Sao Paulo - Hospital São Paulo
102. Comitê de Ética em Pesquisa - Hospital Mãe de Deus
103. Comitê de Ética em Pesquisa da Fundação Antônio Prudente - AC Camargo Câncer Center, Rua Professor Antônio Prudente 211, 01509-900, São Paulo, São Paulo, Brazil
104. Ethics Committee for Multi-Centre Trials; 5 Sveta Nedelya Square Sofia Sofia-Grad 1000 Bulciaria
105. Ethics Committee at Clinical Oncology Dispensary
106. Ethics Committee at City Clinical oncologic dispensary
107. Ethics Committee at Russian Oncology Research Center n.a. N.N.Blokhin
108. Ethics Committee at Moscow City Oncology Hospital #62 of Moscow Healthcare Department
109. Ethics Committee at Volzhskiy regional clinical oncology dispensary #3
110. CEQ of Ivano-Frankivsk Regional Oncology Dispensary
111. CEIC Hospital Universitario Insular Materno-Infantil de Las Palmas
112. CEIC Hospital Universitari Vall d'Hebron
113. CEIC Hospital Universitario Ramón y Cajal
114. CEIC Hospital Universitario 12 de Octubre

115. Etická komisia Prešovského samosprávneho kraja
116. Etická komisia Univerzitna nemocnica Bratislava
117. Lithuanian Bioethics Committee
118. CEIC Hospital Universitari de Bellvitge
119. Etická komisia pri Národnom onkologickom ústave
120. Kaiser Permanente of Colorado Institutional Review Board; 10065 East Harvard Avenue Suite 300 Denver Colorado 80231 United States
121. Ethics Committee at Russian Medical Military Academy n.a. S.M.Kirov
122. Comité de Ética en investigación del instituto Regional de Enfermedades Neoplásicas
123. National Cheng Kung University Hospital Human Experiment and Ethic Committee
124. Houston Methodist Research Institute IRB; 6670 Bertner Suite 6-351 Houston Texas 77030 United States
125. Ingalls Memorial Hospital IRB; 1 Ingalls Drive Ingalls Memorial Hospital Harvev Illinois 60426 United States
126. Rush University Medical Center Institutional Review Board; 1653 West Congress Parkway Chicago Illinois 60612 United States
127. Mercy Health Springfield Communities Institutional Review Board; 1235 East Cherokee Street Springfield Missouri 65804 United States
128. Mayo Clinic Institutional Review Board; 200 First Street Southwest Rochester Minnesota 55905 United States
129. Salus IRB; 2111 West 78758 Braker United Lane States Suite 400 Austin Texas
130. Mackay Memorial Hospital Institutional Review Board
131. Commission on Ethics Questions of Vinnytsya Regional Clinical Oncological Dispensary
132. Comite Etico Cientifico Clinica Santa Maria
133. BRANY IRB; 225 Community Drive Suite 100 Great Neck New York 11021 United States
134. Yale University Human Research Protection Program; 55 College Street New Haven Connecticut 6510 United States
135. Maimonides Med Ctr Institutional Review Board; 4802 10th Avenue Brooklyn New York 11219 United States
136. Scripps Health Institutional Review Board; 11025 North Torrey Pines Road Suite 200 La Jolla California 92037 United States
137. University of Texas Health Science Center San Antonio Institutional Review Board; 7703 Floyd Curl Drive Greyhound North Campus Research Administration Room 2.104 San Antonio Texas 78229 United States
138. Park Nicollet Institute Institutional Review Board; 3800 Park Nicollet Boulevard Minneapolis Minnesota 55416 United States
139. Eticka komisia NsP Sv. Jakuba, n.o., Bardejov
140. Ethikkommission der Bayerischen Landesärztekammer
141. CEQ of Kyiv City Clinical Oncological Center
142. The Institutional Review Board Hospital of China Medical University
143. Ethics Committee at Private Medical Institution "Evromedservis"
144. Comitato di Bioetica dell'AUSL 1 di Sassari
145. Eticka komisia Onkologicky ustav sv. Alzbety
146. Ethics Committee at Railway Clinical Hospital JSC RZD
147. Ethics Committee for Multi-Centre Trials, Bulgaria
148. Sault Area Hospital Research Ethics Board; 750 Great Northern Road Sault Ste. Marie Ontario P6B DAB Canada
149. Toranomon Hospital and Toranomon Hospital Kajigaya IRB, 2-2-2 Toranomon, Minato-ku, 105-8470, Tokyo, JAPAN
150. Kitasato University Sagamihara IRB
151. Niigata Cancer Center Hospital IRB
152. Kyoto University Hospital IRB
153. Osaka City University Hospital IRB
154. National Hospital Organization Toneyama National Hospital IRB
155. Wakayama Medical University IRB
156. Kurume University IRB

157. National Hospital Organization Kyushu Cancer Center; IRB
158. Kanagawa Cardiovascular and Respiratory Center IRB
159. National Hospital Organization Kyushu Medical Center IRB
160. National Hospital Organization Shikoku Cancer Center IRB
161. Miyagi Cancer Center IRB
162. Kyorin University Hospital IRB
163. Center Hospital of the National Center for Global Health and Medicine IRB

IMpower133

1. Hospital Ramón y Cajal ;Comité Ético de investigación Clínica, Ctra. Colmenar Viejo, km 9,1, 28034, Madrid, MADRID, SPAIN
2. Ethikkommission d. Landes Oberösterreich, Wagner-Jauregg-Weg 15, 4020, Linz, AUSTRIA
3. Samsung Medical Center EC, 81, Irwon-ro, Gangnam-gu, 06351, Seoul, KOREA, REPUBLIC OF
4. Seoul National University Hospital; IRB, 101, Daehak-ro, Jongno-gu, 03080, Seoul, KOREA, REPUBLIC OF
5. Asan Medical Center Ethics Committee; Asan Medical Center; IRB
6. National Ethics Committee, Ministry of Health and Social Wellfare, 284, Messogion Avenue, 15562, Cholargos, GREECE
7. Comitato Etico Indipendente della Fondazione IRCCS Istituto Nazionale dei Tumori di Milano, Via Giacomo Veneziani 1, 20133, Milano, Lombardia, ITALY
8. Comitato Etico per Parma, Via Gramsci, 14, 43126, Parma, Emilia-Romagna, ITALY =
9. Università Campus Bio-Medico di Roma, Via Alvaro del Portillo 200, 00128, Roma, Lazio, ITALY
10. COMITATO ETICO DELL'IRCCS GIOVANNI PAOLO II DI BARI PRESSO IRCCS CASA SOLLIEVO DELLA SOFFERENZA, V. le Cappuccini 1, 71013, San Giovanni Rotondo, Puglia, ITALY
11. Comitato Etico Degli IRCCS Istituto Europeo di Oncologia e Centro Cardiologico Monzino, VIA RIPAMONTI 435, 20141, MILANO, Lombardia, ITALY
12. Comitato Etico Regione Toscana - Area Vasta Nord Ovest
13. Copernicus Group IRB, 5000 CentreGreen Way, Suite 200, CARY, NC, 27513, UNITED STATES
14. Copernicus Group IRB, One Triangle Drive, Suite 100, Research Triangle Park, NC, 27709, UNITED STATES
15. Florida Hospital IRB, 901 N. Lake Destiny Rd, Suite 400, Maitland, FL, 32751, UNITED STATES
16. WIRB-Western institutional Review Board, 1019 39th Avenue SE, Suite 120, Puyallup, WA, 98374, UNITED STATES
17. Copernicus Group IRB, 5000 CentreGreen Way, Suite 200, CARY, NC, 27513, UNITED STATES
18. Melbourne Health Human Research Ethics Committee, Flemington Rd, Office for Research Level 6 East, Main Building, 3050, Victoria, Victoria, AUSTRALIA
19. Concord Repatriation General Hospital HREC, Ground Floor -Building 20, Hospital Road, 2139, Concord, New South Wales, AUSTRALIA
20. Lehigh Valley Health Network IRB, 1019 39th Avenue SE, Puyallup, WA, 98374, UNITED STATES
21. Vanderbilt University Institutional Review Board, 1313 21st Ave. South, 504 Oxford House, Nashville, TN, 37232-4315, UNITED STATES
22. Rush University Medical Center; Rush University Research and Clinical Trials Administration Office, 1653 West Congress Parkway, Chicago, IL, 60612-3833, UNITED STATES
23. Western Institutional Review Board, 3535 Seventh Avenue SW, Olympia, WA, 98502, UNITED STATES
24. Chesapeake Research Review; IRB, 7063 Columbia Gateway Drive, Suite 110, Columbia, MD, 21046, UNITED STATES
25. Niezależna Komisja Bioetyczna ds. Sadan Naukowych przy GUMed, ul. M. Skłodowskiej-Curie 3a, 80-210, Gdansk, POLAND
26. Gyogyszereszeti es Egeszsegugyi Minoseg- es Szervezetfejlesztesi Intezet; OGYI, Zrinyi u. 3, H-1051, Budapest, HUNGARY
27. Eticka komise pri IKEM a TN, Videliska 800, 14059, Prague, CZECH REPUBLIC
28. Western Institutional Review Board, 1019 39th Avenue SE, Ste 120, Puyallup, WA, 98374, UNITED STATES
29. Mayo Clinic Institutional Rev Bd Rochester, 200 First Street SW, 201 Building, Room 4-60, Rochester, MN, 55905, UNITED STATES

30. NRES Committee East Midlands - Leicester, The Old Chapel, Royal Standard Place,, Nottingham, NG1 6FS, UNITED KINGDOM
31. Clinical Hospital Center Bezanijska kosa; Ethics Committee Clinical Hospital Center Bezanijska kosa, Bezanijska kosa bb, 11000, Belgrade, SERBIA
32. Ethics Committee Clinical Center Nis, Bulevar Dr Zorana Dindica 48, 18-000, Nis, SERBIA
33. EC at the St. Petersburg City Clinical Oneal. Disp., PROSPEKT VETERANOV, 56, 198255, ST PETERSBURG, RUSSIAN FEDERATION
34. Ethics Committee of the Main Military Clinical Hospital n.a.
35. N.N.Burdenko, 3 Gospitalnaya square, 105229, Moscow, RUSSIAN FEDERATION
36. CITY CLINICAL ONCOLOGY HOSPITAL; Onco, KRASNOGORSKI DISTRICT, p/o Stepanovskoe, 143423, MOSCOW, RUSSIAN FEDERATION
37. Blokhin Russian Cancer Research Center Ethics Committee , Kashirskoye shosse,24, Moscow, RUSSIAN FEDERATION
38. FSBI Research Oncology Institute n.a. N.N.Petrov of Ministry of Health of Russian Federation; onco, Persochny , Leningradskaya Sir., bid. 68, 197758, Saint-Petersburg, RUSSIAN FEDERATION
39. E Cat City Clinical Hospital No 1, Ulitsa Zalesskogo 6, 630047, Novosibirsk, RUSSIAN FEDERATION
40. Etická Komise Fakultni Nemocnice Olomouc, I.P. PAVLOVA 185/6, 779 00, Olomouc, CZECH REPUBLIC
41. Ethics Committee Clinical Center Of Serbia, PASTEROVA 2, 11000, BELGRADE, SERBIA
42. CEP para Análise de Projetos de Pesquisa do HCFMUSP e da FMUSP;Hospital da Universidade de São Paulo, Rua Doutor Arnaldo, 455 - 01246-903, 05403-010, São Paulo, SP, BRAZIL
43. Comitê de Ética do Hospital de Clínicas de Porto Alegre, Rua Ramiro Barcelos, 2350, 90035-903, Porto Alegre, RS, BRAZIL
44. Hospital Santa Izabel - Santa Casa de Misericórdia da Bahia, Pra9a Almeida Couto 500, 40050-410, Salvador, BA, BRAZIL
45. Ethikkommission der Ärztekammer Schleswig-Holstein, Bismarckallee 8-12, 23795, Bad Segeberg, GERMANY
46. Research Ethics Committee, Nat. Taiwan Univ. Hosp., 7 CHUNG-SHAN SOUTH ROAD , 100, TAIPEI, TAIWAN
47. TVGH Institutional Review Board, No.201, Shih-Pai Road, Sec.2, 112, Taipei, TAIWAN
48. Chang Gung Med Found, Institutional Review Board, No. 123, Dunghu Rd., Jioulu Village, Taoyuan County, 333, Gueishan Township, TAIWAN
49. Comité de Ética Servicio de Salud Metropolitano Norte, Maruri 272, Independencia, 8380656, Santiago, CHILE
50. Comité de Ética Servicio de Salud Metropolitano Oriente, Av. Salvador 364, Providencia, 7500922, Santiago, CHILE
51. Seoul National University Bundang Hospital IRB, 82, Gumi-Ro 173 Bean-Gil, Bundang-Gu, 463-707, Seongnam-Si, Gyeonggi-Do, KOREA, REPUBLIC OF
52. EK des Landes Sachsen-Anhalt, Kühnauer Str. 70, 06846, Dessau-Roßlau, GERMANY
53. Ethikkommission der Medizinischen Fakultät Heidelberg, Alte Glockengießerei 11 /1, 69115, Heidelberg, GERMANY
54. Ethik-Kommission der Bayerischen Landesärztekammer, Mühlbaurstr. 16, Sekretariat, 81677, München, GERMANY
55. EK Hessen LAK, Im Vogelsang 3, 60488, Frankfurt, GERMANY
56. CPP Nord Ouest IV, CHU de Lille - Service de Pharmacologie, 1 Place de Verdun, 59045, LILLE, FRANCE
57. Melbourne Health Human Research Ethics Committee, Grattan Street, 3050, PARKVILLE, Victoria, AUSTRALIA
58. Shizuoka Cancer Center Ethical Review Board for Business Clinical Studies, 1007 Shimonagakubo Nagaizumi-cho, Suntou-gun, 411-8777, Shizuoka, JAPAN
59. Kurashiki Central Hospital Institutional Review Board, 1-1-1 Miwa, Kurashiki-shi, 710-8602, Okayama, JAPAN
60. Kindai University Hospital Institutional Review Board, 377-2 Ohnohigashi, Osaka-Sayama-shi, 589-8511, Osaka, JAPAN
61. Sendai Kousei Hospital Institutional Review Board, 4-15 Hirose-Machi, Aoba-Ku, Sendai-shi, 980-0873, Miyagi, JAPAN

62. Wakayama Medical University Institutional Review Board, 811-1 Kimiidera, Wakayama-shi, 641-8510, Wakayama, JAPAN
63. The Cancer Institute Hospital of JFCR Institutional Review Board, 3-8-31 Ariake Koto-Ku, 135-8550, Tokyo, JAPAN
64. Saitama Cancer Center Institutional Review Board, 780 Komuro Inamachi, Kitaadachi-gun, 362-0806, Saitama, JAPAN
65. Kanagawa Cancer Center IRB, 1-1-2, Nakao, Asahi-ku, Yokohama-shi, 241-8515, Kanagawa, JAPAN
66. Kyushu University Hospital IRB, 3-1-1 Maidashi, Higashi-Ku, Fukuoka-Shi, 812-8582, Fukuoka, JAPAN
67. National Hospital Organization Kinki-chuo Chest Medical Center Institutional Review Board, 1180 Nagasone-cho, Kita-ku, Sakai, 591-8555, Osaka, JAPAN
68. Tokyo Metropolitan Komagome Hospital Ethics Committee, 3-18-22 HONKOMAGOME, BUNKYO-KU, 113-8677, TOKYO, JAPAN
69. Institutional Review Board of National Hospital Organization Himeji Medical Center, 68 Honmachi, Himeji-shi, 670-8520, Hyogo, JAPAN
70. University Hospital, Kyoto Prefectural University of Medicine Institutional Review Board, 465 Kajii-cho, Kawaramachi-dori Hirokoji Agaru, Kamigyo-ku, 602-8566, Kyoto, JAPAN
71. CEI de Clinica Bajio CLINBA; Comité de Ética en investigación, Valenciana 7, Col. Paxtitlan. Guanajuato, Gto., 36090, Guanajuato, MÉXICO
72. Ethic Committee of Jilin Cancer Hospital, 1018 HUGUANG ROAD, CHAOYANG DISTRICT, 130012, CHANGCHUN, CHINA
73. EC of Zhongshan Hospital Fudan University, 180 FENGLIN ROAD, 1474 WEST YANAN ROAD, 200032, SHANGHAI, CHINA
74. EC of Fudan University Shanghai Cancer Center, 5th Floor, Building 2, No.270, Dong'an Road, 200032, Shanghai, CHINA
75. EC Of The First Affiliated Hospital Of Guangzhou Medical University, No.151,Yanjiang Road, 510120, Guangzhou, CHINA
76. Henan Tumor Hospital; Ethics committee of Henan Tumor Hospital, 127# Dongming Rd, Jinshui District, 450008, Zhengzhou, CHINA
77. The Ethics Committee of Beijing Cancer Hospital, No.52 Fucheng Road .. Haidian District .. 100036, Beijing, CHINA
78. Harbin Medical University Cancer Hospital; Ethics Committee, No.150, Haping Road, Nangang District, 150081, Harbin, CHINA
79. Zhejiang Cancer Hospital; Ethics Committee/IRS, second floor, building of administration, No.38, Guangji Road, 310022, Hangzhou City, CHINA
80. EC of Jiangsu Cancer Hospital, No.42, Baiziting, 210009, Nanjing, CHINA

IMpower131
1. Comité Institucional de Ética de Investigación en Salud Sanatorio Allende, Hipolito Yrigoyen 384, X5000JHQ, Córdoba, Córdoba, Argentina
2. Comité Institucional de Ética de Investigación en Salud del Instituto Médico Río Cuarto, Hipólito Yrigoyen 1020, 5800, Río Cuarto, Córdoba, Argentina
3. Comité De Ética Del Hospital Provincial Del Centenario, Urquiza 3101, S2002KDS, Rosario, Santa Fe, Argentina
4. Comité de Ética Independiente - Fundación Sanatorio, Francisco Acuña de Figueroa 1240, C1180AAX, Buenos Aires, Ciudad Autónoma de Buenos Aires, Argentina
5. Comite De Etica Del Sanatorio Britanico Sa, Paraguay 40, S2000CVB, Rosario, Santa Fe, Argentina
6. Comité de Ética en Investigación del Instituto Alexander Fleming, Cramer 1180, C1426ANZ, Buenos Aires, Ciudad Autónoma de Buenos Aires, Argentina
7. Comité Independiente de Ética en investigación clínica "Dr. Carlos A. Barclay, Larrea 1381, 3° A, 1117, Buenos Aires, Ciudad Autónoma de Buenos Aires, Argentina
8. Comité de ética en investigación Fundación Oncosalud, Siria 16, 2700, Perga, Buenos Aires, Argentina
9. Comité de Ética Centro de Oncología e Investigación Buenos Aires, Calle 12 #4756, B1880BBF, Berazategui, Buenos Aires, Argentina

10. Comité de Ética Independiente Patagónico, San Martin 391, L6300DVM, Santa Rosa, La Pampa, Argentina
11. Hunter New England Research Ethics and Governance Unit, Locked Bag 1, 2305, New Lambton, New South Wales, Australia
12. Tasmania Health and Medical Human Research Ethics Committee, 301 Sandy Bay Road, 7001, Hobart, Tasmania, Australia
13. Bellberry Human Research Ethics Committee, 123 Glen Osmond Road, 5063, Eastwood, South Australia, Australia
14. Cabrini Human Research Ethics Committee, 183 Wattletree Road, 3144, Malvern, Vic, Australia
15. Sir Charles Gairdner Hospital HREC, Hospital Avenue, 6009, Nedlands, Western Australia, Australia
16. Sydney Local Health District Human Research Ethics Committee - CRGH, Hospital Road, 2139, Concord, New South Wales, Australia
17. Ethikkommission für das Bundesland Salzburg, Sebastian-Stief-Gasse 2, 5010, Salzburg, Salzburg, Austria
18. Ethikkommission des Landes Oberösterreich, Wagner-Jauregg Weg 15, 4020, Linz, , Austria
19. AZ Sint Augustinus, Oosterveldlaan 24, 2610, Wilrijk, Antwerpen, Belgium
20. Comité d'Ethique hospitalo-facultaire Cliniques universitaires Saint-Luc, Promenade de l'Alma 51 bte B1.43.03, 1200, Bruxelles, Brussels, Belgium
21. Ethisch Comité Werken Glorieux VZW, Glorieuxlaan 55, 9600, Ronse, Oost-Vlaanderen, Belgium
22. Comitê de Ética em Pesquisa do Hospital de Clínicas de Porto Alegre, Rua Ramiro Barcelos 2350, 90035-903, Porto Alegre, Rio Grande do Sul, , Brazil
23. Comitê de Ética em Pesquisa da Universidade de Caxias do Sul, Rua Francisco Getúlio Vargas 1130, 95070-560, Caxias do Sul, Rio Grande do Sul, Brazil
24. Comitê de Ética em Pesquisa em Seres Humanos do Centro Universitario UNIVATES/RS, Rua Avelino Tallini 171, Bairro Universitário, 95900-000, Lajeado, Rio Grande do Sul, Brazil
25. Comitê de Ética em Pesquisa da Fundação Antônio Prudente - AC Camargo Câncer Center, Rua Professor Antônio Prudente 211, 01509-900, São Paulo, São Paulo, Brazil
26. Comitê de Ética em Pesquisa - Hospital Mãe de Deus, R. José de Alencar 286, 90880-480, Porto Alegre, Rio Grande do Sul, Brazil
27. Comitê de Ética em Pesquisa em Seres Humanos da Irmandade da Santa Casa de Londrina, Rua Espírito Santo, 523, 86010-510, Londrina, Paraná, Brazil
28. Comitê de Ética em Pesquisa em Seres Humanos da Universidade de Ribeirão Preto (UNAERP), Avenida Costábile Romano, 2201, Ribeirania, 14096380, Ribeirão Preto, São Paulo, Brazil
29. Comitê de Ética em Pesquisa em Seres Humanos do Hospital Lifecenter, Avenida Do Contorno, 4747, 30110-921, Belo Horizonte, Minas Gerais, Brazil
30. Comitê de Ética em Pesquisa Fundação Pio XII Hospital de Câncer de Barretos, Rua Antenor Duarte Villela 1331, 14784-400, Barretos, São Paulo, Brazil
31. Comite de Etica em Pesquisa da Universidade Federal de Sao Paulo - Hospital Sao Paulo, Rua Botucatu 572, 04023-062, São Paulo, , Brazil
32. Comitê de Ética em Pesquisa em Seres Humanos do Hospital Socor, Rua Tupis, 1540, 30190-062, Belo Horizonte, Minas Gerais, Brazil
33. Comitê de Ética em Pesquisa em Seres Humanos da Faculdade de Medicina de São José do Rio Preto, Avenida Brigadeiro Faria Lima 5416, Vila São Pedro, 15090-000, São José Do Rio Preto, , Brazil
34. Ethics Committee for Clinical Trials, 5 Sveta Nedelya Square, 1000, Sofia, Sofia-Grad, Bulgaria
35. Comité d'éthique de la recherche de l'Hôpital Maisonneuve-Rosemont, 5415 Boulevard De L'assomption, H1T 2M4, Montréal, Québec, Canada
36. William Osler Health system Research Ethics Board, 2100 Bovaird Drive East, L6W 3J7, Brampton, Ontario, Canada
37. Royal Victoria Regional Health Centre Research Ethics Board, 201 Georgian Drive, L4M 6M2, Barrie, Ontario, Canada
38. Lakeridge Health REB, 1 Hospital Court, L1G 2B9, Oshawa, Ontario, Canada
39. Centre de Santé et de Services Sociaux de Saint-Jérôme (CSSS) - Comité d'éthique de recherche, 290 Rue de Montigny, J7Z 5T3, Saint Jérôme, Québec, Canada
40. Comité Ético-Científico del Servicio de Salud Metropolitano Oriente (CEC-SSMO), Av. Salvador 364, , Santiago, Región-Metropolitana Santiago, Chile

41. Comité de Ética Científica de Clínica Santa María, Avenida Bellavista 0373, 7520379, Santiago, Región-Metropolitana Santiago, Chile
42. Comité Ético Científico y de Investigación Hospital Clínico Universidad de Chile, Santos Dummont 999, 8380456, Santiago, , Chile
43. CPP Sud-Est II, Groupement Hospitalier Est- 59 Boulevard Pinel, 69500, Bron, , France
44. Landesamt für Gesundheit und Soziales Berlin (LaGeSo) Ethik, Fehrbelliner Platz 1, 10707, Berlin, Berlin, Germany
45. Ethik-Kommission der Ärztekammer Westfalen-Lippe und der Medizinischen Fakultät der WWU Münster, Gartenstraße 210 - 214, 48147, Münster, Nordrhein-Westfalen, Germany
46. Ethik-Kommissionen bei der Ärztekammer Schleswig-Holstein, Bismarckallee 8-12, 23795, Bad Segeberg, Schleswig-Holstein, Germany
47. Ethikkommission der Ärztekammer Nordrhein, Tersteegenstraße 9, 40474, Düsseldorf, Nordrhein-Westfalen, Germany
48. Ethikkommission der Bayerischen Landesärztekammer, Mühlbaurstraße 16, 81677, München, Bayern, Germany
49. Ethik-Kommission der Ärztekammer Sachsen-Anhalt, Am Kirchtor 9, 6108, Halle an der Saale, , Germany
50. Ethik-Kommission bei der Ärztekammer des Saarlandes, Faktoreistraße 4, 66111, Saarbrücken, Saarland, Germany
51. Ethik-Kommission der Ärztekammer Hamburg, Weidestr. 122 b, 22083, Hamburg, Hamburg, Germany
52. Ethik-Kommission der Bayerischen Landesärztekammer, Mühlbaurstr.16, 81677, München, , Germany
53. Ethikkommission an der Universität Regensburg, Landshuter Straße 4, 93047, Regensburg, , Germany
54. Ethikkommission der Landesärztekammer Hessen, Im Vogelsgesang 3, 60488, Frankfurt am Main, Hessen, Germany
55. Ethikkommission der Landesärztekammer Baden-Württemberg, Jahnstraße 40, 70597, Stuttgart, Baden-Württemberg, Germany
56. Ethik-Kommission der Medizinischen Fakultät "Carl Gustav Carus" der Technischen Universität Dresden, Fetscherstraße 74, 01307, Dresden, , Germany
57. Ethik-Kommission der Ärztekammer Westfalen-Lippe und der Medizinischen Fakultät der WWU Münster, Gartenstraße 210 - 214, 48147, Münster, Nordrhein-Westfalen, Germany
58. Galilee Medical Center EC, Western Galilee Hospital POB 21, 22100, Nahariya, , Israel
59. Rambam Medical Center Ethics Committee, 8 Haaliya Hashniya Street, 31096, Haifa, Haifa, Israel
60. Tel Aviv Sourasky EC, 6 Weitzman Street, 6423906, TEL AVIV, , Israel
61. The Chaim Sheba Medical Center EC, Tel Hashomer, 52621, Ramat Gan, , Israel
62. Shamir Medical Center Assaf Harofeh EC, Beer Yaakov 70300, 70300, Zerifin, , Israel
63. Soroka University Medical Center Local EC, Reger Avenu, 84101, Beer Sheva, , Israel
64. Rabin Medical Center Ethics Committee, 39 Jabotinski St., 49100, Petach Tikva, , Israel
65. Hadassah University Hospital Local EC, Kiryat Hadassah, 91120, Jerusalem, , Israel
66. Kaplan Medical Center Local EC, Kaplan Medical Center, 76100, Rehovot, , Israel
67. Meir EC, 59 Tchernichovsky Street, 44281, Kfar Saba, HaMerkaz, Israel
68. Comitato Etico Regionale Toscana – Area Vasta Nord Ovest, Via Roma 67, 56126, Pisa, Toscana, Italy
69. Comitato Etico Campania Nord, VIA DEGLI IMBIMBO 10-12, 83100, Avellino, Camparia, Italy
70. Comitato Etico San Martino IST 2, Largo Rosanna Benzi 10, 16132, Genova, , Italy
71. Comitato di Bioetica Fondazione IRCCS Policlinico S. Matteo di Pavia, Viale Golgi 19, 27100, Pavia, , Italy
72. Comitato Etico IRCCS
73. Istituto Nazionale per lo Studio e la Cura dei Tumori
74. Fondazione G Pascale, Via Mariano Semmola, 80131, Napoli, Campania, Italy
75. Comitato Etico Catania 1, VIA SANTA SOFIA 78, 95123, Catania, Sicilia, Italy
76. Comitato Etico delle Aziende Sanitarie dell’Umbria, Via della Rivoluzione, 16, 06070, Ellera di Corciano, Perugia, Italy

77. Comitato Etico Area 5 presso IRCCS Ospedale Oncologico di Bari Istituto Tumori Giovanni Paolo II, Viale Orazio Flacco 65, 70124, Bari, Puglia, Italy
78. Comitato Etico dell'Università Federico II, Via Sergio Pansini, 5, 80131, Napoli, Napoli, Italy
79. Comitato Etico Azienda Ospedaliera Universitaria Maggiore della Carità, Corso Mazzini 18, 28100, Novara, Piemonte, Italy
80. Comitato Etico Lazio 1, Circonvallazione Gianicolense, 87, 152, Roma, Lazio, Italy
81. Comitato Etico Cardarelli-Santobono, Via Antonio Cardarelli 9, 80131, Napoli, Campania, Italy
82. The Ethics Committee for Clinical Trials of Medicinal Products, Aizkraukles Street 21-113, LV-1006, Riga, , Latvia
83. Lithuanian Bioethics Committee, Vilniaus str. 16, LT-01402, Vilnius, , Lithuania
84. Comité de Ética en Investigación de México Centre for Clinical Research SA de CV, Amores 709, 3100, Ciudad de México, Distrito Federal, Mexico
85. Comité de Ética en Investigación de la Facultad de Medicina de la UANL y Hospital Universitario "Dr., Av. Francisco I. Madero y Gonzalitos S/N, Colonia Mitras Centro, 64460, Monterrey, Nuevo León, México
86. METC azM/UM, Oxfordlaan 10, 6202 AZ, Maastricht, , Netherlands
87. METC Noord Holland, Nassauplein 10, 1815 GM, Alkmaar, , Netherlands
88. Medical Research Ethics Committees United, Koekoekslaan 1, 3435 CM, Nieuwegein, Utrecht, Netherlands
89. Comité de Ética en Investigación del Instituto Regional de Enfermedades Neoplásicas, Panamericana Norte Km. 558, 12345, Trujillo, , Perú
90. Asociacion Benefica Prisma, Calle Carlos Gonzles 251, Lima 32, Lima, Lima, Peru
91. Comité de Ética en Investigación del Hospital Guillermo Almenara Irigoyen, Avenida Grau 800, Lima 13, Lima, Lima, Peru
92. Comité Institucional de ética en Investigación Instituto Nacional de Enfermedades Neoplásicas, Avenida Angamos Este 2520, Lima 34, Lima, Lima, Peru
93. Comissão de Ética para a Investigação Clínica - CEIC, Avenida do Brasil, 53, 1749-004- Lisboa, Lisboa, Portugal
94. Ethics Committee at Moscow City Oncology Hospital #62 of Moscow Healthcare Department, Krasnogorskiy district, Stepanovskoe, settlement Istra, 27, 143423, Moscow, , Russian Federation
95. Ethics Committee at Russian Medical Military Academy n.a. S.M.Kirov, Ulitsa Academika Lebedeva, 6, 194044, St. Petersburg, , Russian Federation
96. Ethics Committee at Russian Oncology Research Center n.a. N.N.Blokhin, Kashirskoe Shosse 24, 115478, Moscow, , Russian Federation
97. Ethics Committee at Clinical Oncology Dispensary, Ulitsa Zavertyayeva, 9 - 1, 644013, Omsk, , Russian Federation
98. Ethics Committee at City Clinical oncologic dispensary, Vtoraya Beryozovaya Alleya 3/5, 197022, St. Petersburg, , Russian Federation
99. Ethics Committee at Volzhskiy regional clinical oncology dispensary #3, Ulitsa Komsomolskaya, 25, 404100, Volzhskiy, , Russian Federation
100. Domain Specific Review Board, Nexus@One-North (South Tower), 138543, Singapore, , Singapore
101. Singhealth Centralised Institutional Review Board, 7 Hospital Drive, Singhealth Office Of Research, Blk A, #03-01, Singhealth Research Facilities, 169611, Singapore, Singapore, Singapore
102. Etická komisia pri Národnom onkologickom ústave, Klenova 1, 833 01, Bratislava, , Slovakia
103. Etická komisia Univerzitna nemocnica Bratislava, Ruzinovska 6, 826 06, Bratislava, , Slovakia
104. Etická komisia Prešovského samosprávneho kraja, Namestie Mieru 2, 080 01, Presov, , Slovakia
105. CEIC de la Corporacion Sanitaria del Parc Tauli, Calle Parc Tauli, s/n, 8208, Sabadell, Barcelona, Spain
106. CEIC Hospital Universitari de Bellvitge, C/ Feixa Llarga s/n, 8907, L´Hospitalet de Llobregat, Cataluña, Spain
107. CEIC Hospital Universitario Insular Materno-Infantil de Las Palmas, Avenida Marítima del Sur, s/n, 35016, Las Palmas de Gran Canaria, , Spain
108. CEIC Consorcio Hospital General Universitario de Valencia, Avenida Tres Cruces, 2, 46014, Valencia, Valencia, Spain

109. CEIC Hospital Universitario 12 de Octubre, Avenida de Córdoba, s/n, 28041, Madrid, Madrid, Spain
110. CEIC Hospital Universitario Ramón y Cajal, Carretera de Colmenar km. 9.100, 28034, Madrid, Madrid, Spain
111. CEIC Hospital Universitario Vall d'Hebrón, Passeig de la Vall d'Hebron, 119-129, 8035, Barcelona, , Spain
112. CEIC Hospital Clínic de Barcelona, Calle Villarroel, 170, 8036, Barcelona, Barcelona, Spain
113. CEIC de Galicia (CAEI), Edificio Administrativo San Lázaro, s/n, 15781, Santiago de Compostela, A Coruña, Spain
114. CEIC Islas Baleares (CEIC-IB), Camí de Jesús, 38 A, 7011, Palma de Mallorca, Baleares, Spain
115. CEIC Parc de Salut Mar, Calle Doctor Aiguader, 88, 8003, Barcelona, Barcelona, Spain
116. CEIC Hospital Universitario de Canarias, Calle Ofra, s/n - Planta -2, 38320, La Laguna, Santa Cruz de Tenerife, Spain
117. CEIC de Cantabria, Avenida Cardenal Herrera Oria, s/n, 39011, Santander, Cantabria, Spain
118. CEIC Hospital Clínico Universitario de Valencia, Avenida Vicente Blasco Ibánez, 17, 46010, Valencia, Valencia, Spain
119. CEIC Grupo Hospital de Madrid, Avenida Montepríncipe, 25, 28660, Boadilla del Monte, Madrid, Spain
120. CEIC Hospital Clinico San Carlos, Calle Profesor Martin Lagos, s/n, 28040, Madrid, Madrid, Spain
121. CEIC Hospital General Universitario Gregorio Marañon, Calle Doctor Esquerdo, 46, 28007, Madrid, Madrid, Spain
122. CEIC Fundación Jiménez Díaz, Avenida Reyes Católicos, 2, 28040, Madrid, Madrid, Spain
123. CEIC Hospital Universitario La Paz, Paseo de la Castellana, 261, 28046, Madrid, Madrid, Spain
124. CEIC de Andalucia (CCEIBA), Avenida de la Innovación s/n, 41020, Sevilla, Andalucía, Spain
125. CEIC de Aragón (CEICA), Avenida San Juan Bosco, 13, 50009, Zaragoza, Zaragoza, Spain
126. CEIC Hospital Santa Creu i Sant Pau, Avenida Sant Antoni Maria Claret, 167, 8025, Barcelona, Barcelona, Spain
127. Kantonale Ethikkommission Bern (KEK), Murtenstraße 31, 3010, Bern, , Switzerland
128. Mackay Memorial Hospital Institutional Review Board, No.92, Section2, Chung-shan North Road, 104, Taipei, , Taiwan, Province of China
129. Chang Gung Medical Foundation, 199 Tung Hwa North Road, 10507, Taipei, , Taiwan, Province of China
130. Institution Review Board of National Taiwan University Hospital, No.1, Changde-de Street, Zhongzheng Dist, 100, Taipei, , Taiwan, Province of China
131. Institutional Review Board Taipei Veterans General Hospital, No. 201, Sec.2 Shipei Road, Beitou Dist., 11217, Taipei, , Taiwan, Province of China
132. Cheng-Hsin General Hospital Institutional Review Board, 1F, No. 45, Chenghsin Street, Beitou District, 112, Taipei City, , Taiwan, Province of China
133. Institutional Review Board of Tri-Service General Hospital, No.325,Section 2, Cheng-Kung Road, 11490, Taipei, , Taiwan, Province of China
134. Institutional Review Board, Changhua Christian Hospital, No.135 Nansiao Street, 50006, Changhua, , Taiwan, Province of China
135. Institutional Review Board Kaohsiung Medical University Chung-Ho Memorial Hospital, No.100, Tzyou 1st Road, 807, Kaohsiung City, , Taiwan, Province of China
136. The Institutional Review Board of Taichung Veterans General Hospital, No.160 Section 3 Chung-Kang Road, 40705, Taichung, , Taiwan, Province of China
137. Institutional Review Board of the Chi Mei Medical Center, 4F, 3rd Medical building, No. 901 Chung-Huwa Rd., Young-Kang Dist. Tainan, Taiwan., , Tainan, , Taiwan, Province of China
138. China Medical University and Hospital Research Ethics Committee, No.2, Yuh-Der Road, 40447, Taichung, , Taiwan, Province of China
139. CEQ of Regional Municipal Institution Sumy Regional Clinical Oncology Dispensary, Vulytsya Pryvokzalna 31, 40005, Sumy, , Ukraine
140. CEQ of Municipal Noncommercial Institution Regional Center of Oncology, Vulytsya Lisoparkivska 4, 61070, Kharkiv, , Ukraine

141. CEQ of SI Institute of Medical Radiology n.a. S.P. Hryhoriev of NAMS of Ukraine, 82 Pushkinska str., 61024, Kharkiv, , Ukraine
142. CEQ of Lviv State Oncology Regional Treatment Diagnostic Center, 2-A Yaroslava Hasheka str., 79031, Lviv, , Ukraine
143. CEQ of Transcarpathian Regional Clinical Oncology Dispensary, Vulytsya Brodlakovycha, 2, 88014, Uzhgorod, , Ukraine
144. Commission of Ethics Questions on the basis of the Chernivtsi Regional Clinical Oncology Dispensary, Vulytsya Chervonoarmiyska 242, 58013, Chernivtsi, , Ukraine
145. Commission on Ethics Questions of MNPE Central City Clinical Hospital of Uzhhorod City Council, Vulytsya Gryboedova 20, 88000, Uzhgorod, , Ukraine
146. CEQ of Poltava Regional Clinical Oncology Dispensary of Poltava Regional Council, 7a, Volodarskoho Str., 36021, Poltava, , Ukraine
147. LEC of Municipal Non-profit Enterprise "City Clinical Hospital # 4" of Dnipro City Council, Vulytsya Blyzhnya 31, 49102, Dnipropetrovsk, Dnipropetrovs'ka Oblast , Ukraine
148. CEQ of Treatment and Prevention Institution Volyn Regional Oncology Dispensary, Vulytsya Tymiryazeva 1, 43018, Lutsk, , Ukraine
149. CEQ of MI Kryvyi Rih Oncology Dispensary of Dnipropetrovsk Regional Council, 41 Dnipropetrovske Road, 50048, Kryvyi Rih, , Ukraine
150. CEQ of MI of Zaporizhzhia Regional Council Zaporizhzhia Regional Clinical Oncology Dispensary, 177-A Kulturna str., 69040, Zaporizhzhia, Zaporiz'ka Oblast, Ukraine
151. Commission on Ethics Questions of Vinnytsya Regional Clinical Oncology Dispensary, 84 Khmelnytskyy prospekt, 21029, Vinnytsya, , Ukraine
152. University of Nevada Reno Biomedical Institutional Review Board, 1664 North Virginia Street, 89557, Reno, Nevada, United States
153. Copernicus Group Independent Review Board, 1 Triangle Drive, 27709, Research Triangle Park, North Carolina, United States
154. Pinnacle Health Hospitals Institutional Review Board, 205 South Front Street, 17104, Harrisburg, Pennsylvania, United States
155. Western Institutional Review Board, 1019 39th Avenue Southeast, 98374, Puyallup, Washington, United States
156. Kaiser Permanente Northern California Institutional Review Board, 1800 Harrison Street, 94162, Oakland, California, United States
157. University Of Miami, 1500 N.w. 12th Avenue, 33136, Miami, Florida, United States
158. US Oncology Inc. Institutional Review Board, 10101 Woodloch Forest, 77380, The Woodlands, Texas, United States
159. University of Arkansas IRB, 4301 W. Markham Street, 72205, Little Rock, Arkansas, United States
160. Mercy Saint Vincent Medical Center Institutional Review Board, 2213 Cherry Street, 43608, Toledo, Ohio, United States
161. Oregon Health & Science University IRB, 3181 S.W. Sam Jackson Park Road, 97239-3098, Portland, Oregon, United States
162. WIRB Copernicus Group, 1 Triangle Drive, 27709, Research Triangle Park, North Carolina, United States
163. Siouxland Institutional Review Board, 230 Nebraska Street, 51101, Sioux City, Iowa, United States
164. University of Chicago Hospitals Institutional Review Board, 5751 South Woodlawn Avenue, 60637, Chicago, Illinois, United States
165. Lahey Clinic, Inc. Institutional Review Board, 41 Mall Road, 1805, Boston, Massachusetts, United States
166. New England Institutional Review Board, 85 Wells Avenue, 2459, Newton, Massachusetts, United States
167. Ochsner Clinic Foundation Institutional Review Board, 1514 Jefferson Highway, 70121, New Orleans, Louisiana, United States
168. Kaiser Permanente Southern California Institutional Review Board., 393 E. Walnut, 91188, Pasadena, California, United States

169. North Mississippi Health Services, 830 South Gloster Street, 38801, Tupelo, Mississippi, United States
170. Saint Luke's Hospital Institutional Review Board, 915 East First Street, 55805, Duluth, Minnesota, United States
171. Springfield Committee for Research Involving Human Subjects (SCRIHS), 801 North Rutledge Street, 62702, Springfield, Illinois, United States
172. St. Joseph Mercy Health System Institutional Review Board #2 - Oncology Central IRB, 5301 East Huron River Drive, 48106, Ann Arbor, Michigan, United States
173. Loyola University Institutional Review Board, 2160 South First Avenue, 60153, Maywood, Illinois, United States
174. W.G. 'Bill' Hefner VA Medical Center, 1601 Brenner Avenue, 28144, Salisbury, North Carolina, United States
175. Saint Luke's Hospital Institutional Review Board, 4401 Wornall Road, 64111, Kansas City, Missouri, United States
176. St. Luke’s Hospital & Health Network IRB, 801 Ostrum Street, 18015, Bethlehem, Pennsylvania, United States
177. Advarra Institutional Review Board, 6940 Columbia Gateway Drive, 21046, Columbia, Maryland, United States
178. Lancaster General Hospital IRB, 555 North Duke Street, 17604, Lancaster, Pennsylvania, United States
179. Walter Reed National Military Medical Center IRB, 503 Robert Grant Avenue, 20910-7500, Silver Spring, Maryland, United States
180. Copernicus Group Independent Review Board, 1 Triangle Drive, 27709, Research Triangle Park, North Carolina, United States
181. Copernicus Group Independent Review Board, 5000 CentreGreen Way, 27513, Cary, North Carolina, United States
182. St Charles Medical Center, 2500 Northeast Neff Road, 97701, Bend, Oregon, United States

IMpower130

1. Cliniques Universitaires Saint-Luc - Comite Comite d'ethique de la recherche de I'Hôpital Maisonneuve-Rosemont
2. McGill University Health Center Montreal Hospital
3. Royal Victoria Regional Health Centre Research Ethics Board
4. UBC BCCA Research Ethics Board
5. William Osler Health system Research Ethics Board CPP Est III
6. Ethikkommission der Landesärztekammer Baden-Württemberg
7. Ethik-Kommission der Sächsischen Landesärztekammer
8. Joint Chinese University of Hong Kong - New Territories East Cluster Clinical Research Ethics Assaf Harofe Medical Center EC
9. Galilee Medical Center EC
10. Hadassah University Hospital Local EC
11. Kaplan Medical Center Local EC
12. Meir EC
13. Rabin Medical Center Ethics Committee
14. Rabin Medical Center Local EC
15. Rambam Medical Center Ethics Committee Soroka University Medical Center Local EC
16. Tel Aviv Sourasky EC
17. The Chaim Sheba Medical Center EC
18. Comitato Etico Campania Nord
19. Comitato Etico delle Aziende Sanitarie dell'Umbria
20. Comitato Etico delle Province di Chieti e Pescara Comitato Etico IRCCS
21. Istituto Nazionale per lo Studio e la Cura dei Tumori Fondazione G Pascale
22. Comitato Etico Regionale delle Marche
23. Comitato Etico Seconda Università degli Studi di Napoli Az. Osp. Univ. S.U.N. - A.O.R.N. "Ospedali CEIC Consorcio Hospital General Universitario de Valencia
24. CEIC de Aragón (CEICA)

25. CEIC de Galicia (CAEI)
26. CEIC Hospital de la Santa Creu i Sant Pau
27. CEIC Hospital Santa Creu i Sant Pau
28. CEIC Hospital Universitario de Canarias
29. Appalachian Regional Healthcare IRB
30. Banner MD Anderson Cancer Center IRB
31. Biomedical Research Alliance of New York LLC Institutional Review Board
32. Birmingham Veterans Administration Medical Center IRB
33. Copernicus Group Independent Review Board
34. Copernicus IRB
35. Duke University Health System Institutional Review Board
36. Englewood Hospital and Medical Center
37. Kaiser Permanente Northern California Institutional Review Board
38. Kaiser Permanente of Colorado Institutional Review Board
39. Lahey Clinic, Inc. Institutional Review Board Lancaster General Hospital IRB
40. Loyola University Institutional Review Board
41. Mayo Clinic Institutional Review Board
42. MD Anderson Institutional Review Board
43. New England Institutional Review Board
44. North Mississippi Health Services
45. NYU School of Medicine Institutional Review Board Ochsner Clinic Foundation Institutional Review Pinnacle Health Hospitals Institutional Review Rhode Island Hospital Institutional Review Board
46. Saint Barnabas Medical Center Institutional Review Board
47. Siouxland Institutional Review Board
48. Springfield Committee for Research Involving Human Subjects (SCRIHS)
49. The Christ Hospital IRB
50. University of Arkansas IRB
51. University of Chicago Hospitals Institutional Review Board
52. University Of Iowa Human Subjects Office IRB University of Louisville IRB
53. University Of Miami
54. University of Nevada Reno Research Integrity Office
55. W.G. 'Bill' Hefner VA Medical Center
56. Walter Reed National Military Medical Center IRB
57. Western Institutional Review Board (WIRB)
58. WIRB Copernicus Group

IMpassion130

1. Centro de Pesquisas Oncológicas – CEPON
2. Comitê de Ética em Pesquisa do; HCPA, Rua Ramiro Barcelos
3. Comitê de Ética em Pesquisa da PUCRS, Avenida Ipiranga
4. Etická Komise Fakultni Nemocnice Olomouc
5. Komisja Bioetyczna przy instytucie Centrum Onkologii w
6. Peter Maccallum Cancer Centre Ethics Committee
7. Bellberry Human Research Ethics Committee
8. St John of God Health Care Ethics Committee
9. Research Ethics Committee, Nat. Taiwan Univ. Hosp.
10. Institutional Review Board, Taipei Veterans General Hospital
11. Asan Medical Center Ethics Committee
12. Seoul National Univ. Ethics Committee
13. SeveranceHospital- YonseiUniversity; IRB
14. Samsung Medical Center EC
15. CEP da Real e Benemerita Associacao Portuguesa de Beneficência/SP
16. E. C. of SI of Healthcare Kazan Oncology Dispensary
17. Ivanovo Regional Clinical Oncology Dispensary Ethics Committee
18. LEC of Arkhangelsk' regional clinical oncology dispensary
19. EC of Moscow City Oncol. Hospital #62

20. Comité de Ética del Centro Oncológico y de investigaciones Buenos Aires (CECOIBA)
21. Comité de Ética en Investigación Clínica (CEIC)
22. Institut Jules Bordet, Comité d'Ethique
23. Ontario Cancer Research Ethics Board
24. UBC BCCA Research Ethics Board (BCCA REB)
25. Comité d'éthique de la recherche, Hopital Saint Sacrement du CHA
26. Health Research Ethics Board of Alberta
27. Nova Scotia Health Authority Research Ethics Board
28. Lakeridge Health Research Ethics Board
29. EK Lubeck
30. EK Essen Ethikkommission der Medizinischen Fakultat der Universitat Duisburg-Essen
31. Ethikkommission Landesärztekammer Rheinland-Pfalz
32. Ethik-Kommission am Universitätsklinikum Carl-Gustav-Carus Technische Universität Dresden
33. Ethikkommission Technische Universität München, Fakultät für Medizin
34. EK an der Med. Fakultat d. Eberhard-Karls-Uni und am Uniklinikum Tübingen
35. Ethikkommission der Medizinischen Fakultät Heidelberg
36. Ethik-Kommission der Medizinischen Fakultat der Friedrich-Alexander-Universitat Erlangen-Nurnberg
37. NRES Committee London - City and East; Bristol Research Ethics Committee Centre
38. Stanford Research Compliance Office
39. MedStar Health Research Institute-Georgetown Univ. Oncology IRB
40. Vanderbilt University Institutional Review Board
41. NYU Langone Medical Center IRB
42. UCSF Committee on Human Research
43. US Oncology IRB
44. Yale University Human Research Protection Program
45. UT MD Anderson Cancer Centre
46. Comitato Etico IRCCS Pascale
47. Comitato Etico Istituto Europeo Oncologico
48. QUORUM REVIEW IRB
49. Kaiser Permanente Northern California
50. Pirkanmaan sairaanhoitopiirin eettinen toimikunta
51. Providence Health System IRB
52. West Michigan Cancer Center IRB
53. Johns Hopkins Medicine Institutional Review Board
54. Wayne State University Human Investigation committee
55. Kaiser Permanente Southern California
56. Mercy Medical Center IRB
57. Norwalk Hospital IRB
58. EK der Ärztekammer Nordrhein
59. Ethik-Kommission Berlin Landesamt für Gesundheit und Soziales
60. Ethik-Kommission der Arztekammer Hamburg
61. Ethik-Kommission der Arztekammer Westfalen-Lippe und der Medizinischen Fakultät der WWU Münster
62. Kaiser Permanente Colorado Institutional Review Board
63. WIRB-Western Institutional Review Board
64. Mount Sinai Medical Center IRB
65. Biomedical Research Alliance of New York
66. Wellmont Health System Institutional Review Board
67. Ethikkommission der Universität Wien /AKH
68. Institut Jules Bordet, Comité d'Éthique
69. Hacettepe University Ethics Committee; Ethics Committee
70. Ethikkommission der Martin-Luther-Universität Halle-Wittenberg
71. Clinical Hospital Center Bezanijska kosa; Ethics Committee
72. HKU/HA HKW IRB
73. National Ethics Committee
74. Regionala Etikprövningsnämnden i Uppsala

75. REK Vest; Universitetet i Bergen, Det Medisinske Fakultet
76. Ontario Cancer Research Ethics Board,
77. The Methodist Hospital Research Institute IRB
78. Ethical Clearance Committee on Human Rights
79. Ethics Committee, Faculty of Medicine, Siriraj Hospital
80. Research Ethics Committee China Medical University & Hospital
81. Kaohsiung Medical University Hospital, IRB,
82. Ethikkommission Nordwest- und Zentralschweiz (EKNZ)
83. Ethikkommission Ostschweiz (EKOS)
84. Kantonale Ethikkommission Zürich (KEK)
85. Ethic committee of Clinical Center University of Sarajevo
86. Ethics Committee of Kyiv City Oncological Hospital,
87. Ethics Committee of Dnipropetrovsk City Multilat.
88. Local Ethics Committee of Lviv State Reg. Oncol. Med. Diagn. Centre,
89. Ethics Committee of Treatment and Preventive Institution "Volyn Regional Oncology Dispensary"
90. S-Pb clinical scientific practical center of specialized kinds of medical care (oncological); Center, Pesochnie
91. Ethics Committee of the Main Military Clinical Hospital n.a. N.N.Burdenko
92. Hacettepe University Ethics Committee
93. Comitê de Ética em Pesquisa; Universidade Regional do Noroeste do Estado do Rio Grande do Sul UNIJUÍ
94. Comite de Etica em Pesquisa do Centro de Referencia da Saude da Mulher
95. Comitê de Ética do Hospital de Clínicas de Porto Alegre
96. Comitê de Ética em Pesquisa Seres Humanos da Universidade Federal do Ceará
97. Comité de Ética en investigación Clínica (CEIC)
98. Instituto Conmemorativo Gorgas de Estudios de la Salud Comité de Bioética de la investigación
99. Comite de Etica Independiente Zugueme
100. Comité Ético Científico Universidad de Ciencias Medicas, Sabana Norte
101. Comité de Ética en investigación Sanatorio Alcocer Pozo S.A de C.V.
102. Komisja Bioetyczna przy Instytucie Centrum Onkologii w Warszawie
103. Etická Komise pro Multicentrická klinická hodnoceni; Fakultni Nemocnice v Motola
104. Tallinn Medical Research Ethics Committee, National Institute for Health Development
105. Medical Research Council, Ethics Committee for Clinical Pharmacology
106. Ethics Committee for Clinical Trials of Medicinal Products
107. Republic of Slovenia National Medical Ethics Committee
108. Comité de Ética en investigación de Mexico Centre for Clinical Research SA de CV,
109. Bellberry Human Research Ethics Committee, Bellberry Limited
110. Comisia Națională de Bioetică a Medicamentului și a Dispozitivelor Medicale
111. Instituto Estatal de Cancerología de Colima; Comité de Ética en investigación
112. Comité de Ética en investigación de Mexico Centre for Clinical Research SA de CV
113. SingHealth Centralised IRB; Review Board B,
114. Comité De Ética De investigación Clínica
115. Comité de Ética Científico del Servicio de Salud Araucanía Sur
116. CEIC Pare de Salut Mar; IMIM- Hospital del Mar, Cl Dr. Aiguader,
117. NRES Committee London - City and East
118. Comite De Etica Medica e Investigacion
119. Eticka komise pro multicentricka klinicka hodnocen
120. CPP Sud Est IV, Centre Leon Berard
121. QUORUM REVIEW IRB,
122. St. Luke's Hospital and Health Network IRB
123. Washington University Medical Center, Human Studies Committee
124. Quorum
125. Rush University Medical Center; Rush University Medical Center Institutional Review Board
126. Kaiser Permanente Northwest Region IRB
127. National Cancer Center Institutional Review Board KOREA, REPUBLIC OF
128. Sydney Local Health District Ethics Review Committee (RPAH Zone)

129. Comite de Etica de la Pontificia Uni Catolica de Chile
130. CEI del Instituto Tecnológico y de Estudios Superiores de Monterrey
131. Ethikkommission der Ärztekammer Niedersachsen, Berliner Allee
132. Hiroshima University hospital Institutional Review Board
133. National Hospital Organization Hokkaido Cancer Center IRB
134. Tohoku University Hospital Institutional Review Board
135. Fukushima Medical University Hospital Institutional Review Board
136. Gunma Prefectural Cancer Center Institutional Review Board
137. Saitama Cancer Center Institutional Review Board
138. Saitama Medical University International Medical Center IRB
139. National Cancer Center Institutional Review Board
140. The Cancer Institute Hospital of JFCR Institutional Review Board
141. Tokyo Metropolitan Komagome Hospital Ethics Committee
142. Tokai University Hospital Institutional Review Board
143. IRB of a group of St. Marianna Univ. School of Medicine Hospitals,
144. Niigata Cancer Center Hospital Institutional Review Board
145. Shizuoka Cancer Center Ethical Review Board for Clinical Studies
146. Aichi Cancer Center Hospital Institutional Review Board
147. Mie University Hospital Institutional Review Board
148. Kyoto University Hospital Institutional Review Board
149. Institutional Review Board of Osaka International Cancer Institute
150. Kindai University Hospital Institutional Review Board
151. National Hospital Organization Osaka National Hospital IRB
152. Hyogo College of Medicine Institutional Review Board,
153. Hiroshima City Hospital Institutional Review Board,
154. Sagara Hospital Institutional Review Board
155. Okinawa Medical Association Institutional Review Board
156. The Institutional Review Board of Kumamoto University Hospital
157. St. Luke's International Hospital Institutional Review Board
158. IRB of Okayama University Hospital
159. NRES Committee London - City and East Bristol Research Ethics Committee Centre
160. Bristol Research Ethics Committee Centre
161. Comitê de Ética em Pesquisa Associacao de Combate ao Câncer
162. Blokhin Russian Cancer Research Center Ethics Committee

IMmotion151

1. Macquarie University Human Research Ethics Committee
2. St John of God Health Care Ethics Committee
3. Austin Health HREC, Research Ethics Unit
4. Bellberry Human Research Ethics Committee
5. Concord Repatriation General Hospital HREC
6. Austin Health; Austin Health Human Research Ethics Committee
7. Ethics Committee University Clinical Centre of the Republic of Srpska
8. CEP PUCRS
9. CEP ISCMPOA
10. Comitê de Ética em Pesquisa; Universidade Regional do Noroeste do Estado do Rio Grande do Sul UNIJUi
11. CEP para Análise de Projetos de Pesquisa do HCFMUSP e da
12. FMUSP;Hospital da Universidade de São Paulo
13. Ontario Cancer Research Ethics Board
14. Lakeridge Health Research Ethics Board
15. McGill University; Sir Mortimer B Davis Jewish General Hospital; Ethics Board
16. Nova Scotia Health Authority Research Ethics Board
17. Etická komise Fakultni nemocnice Olomouc
18. Eticka komise Masarykuv onkologicky ustav v Brne
19. Eticka komise pri IKEM a TN, Thomayerova nemocnice

20. Etická komise Všeobecné fakultní nemocnice v Praze
21. Videnskabsetiske Komite Region Midt; Sundhedssekr. Videnskabsetiske Komite Region Midt; Sundhedssektor. Videnskabsetiske Komite Region Midt; Sundhedssekr.
22. CPP lie de France VII
23. Ethik- Kommission der Medizinischen Fakultät Heidelberg
24. Geschäftsstelle der Ethikkommission der Medizinischen Fakultat der Universitat Duisburg- Essen
25. Ethik- Kommission an der Medizinischen Fakultat der Eberhard- Karls-Universität und am Universitätsklinikum Tübingen
26. Ethik- Kommission der medizinischen Fakultat der Ludwig-Maximilians-Universitat
27. Comitato Etico Area Vasta Sud Est
28. Comitato Etico Di Area Vasta Romagna E Irst
29. Comitato Etico Provinciale Modena
30. Comitato Etico dell' Azienda osped. A. Cardarelli
31. Comitato Etico A.O. S. Camillo Forlanini
32. Comitato Etico Irccs Ospedale San Raffaele
33. Comitato Etico Dell'irccs San Matteo Di Pavia
34. Comitato Etico Milano Area C C/0 A.O.Ospedale Niguarda Ca' Granda Nagoya university Hospital IRB
35. Chiba Cancer Center Institutional Review Board
36. Institutional Review Board of Osaka International Cancer Institute Iwate Medical University Institutional Review Board
37. Osaka University Hospital Institutional Review Board
38. Tokushima University Hospital Institutional Review Board
39. The Institutional Review Board of Kumamoto University Hospital
40. The Cancer Institute Hospital of JFCR Institutional Review Board Niigata University Medical & Dental Hospital IRB
41. Hokkaido University Hospital Institutional Review Board
42. Tokyo Women's Medical University Hospital IRB
43. Nippon Medical School Hospital IRB
44. Kindai University Hospital Institutional Review Board
45. University of Tsukuba Hospital IRB
46. Keio University Hospital IRB
47. Yokohama City University Hospital IRB
48. Toranomon Hospital and Toranomon Hospital Kajigaya Institutional Review Board
49. Osaka City University Hospital IRB
50. Okayama University Hospital Institutional Review Board
51. Kyushu University Hospital IRB
52. Kitasato University Sagamihara Institutional Review Board
53. Medical Hospital, Tokyo Medical and Dental University Institutional Review Board
54. Gunma University Hospital Institutional Review Board
55. Seoul National University Hospital; IRB
56. Severance Hospital Yonsei University; IRB
57. Samsung Medical Center, IRB
58. Asan Medical Center Ethics Committee; Asan Medical Center; IRB
59. Seoul National University Bundang Hospital IRB
60. National Cancer Center Institutional Review Board KOREA
61. Chungnam National University Hospital; IRB
62. CEI de Clinica Bajio CLINBA; Comité de Ética en investigación
63. Komisja Bioetyczna przy Uniwersytecie Medycznym im. K. Marcinkowskiego. Komisja Bioetyczna przy Uniwersytecie Medycznym im. K. Marcinkowskiego. Komisja Bioetyczna przy Uniwersytecie Medycznym im. K. Marcinkowskiego. Komisja Bioetyczna przy Uniwersytecie Medycznym im. K. Marcinkowskiego. EC of Moscow City Oneal. Hospital #62
64. EC at FSI MSROI n.a. Hertsen of Rosmedtechnology
65. EC of FSBI Privolzhsky Federal Medical Research Centre
66. E.C. of Altai Oncological Center
67. SingHealth Centralised IRB; Review Board B

68. CEIC Hospital Vall D'Hebron
69. CEIC Hospital Universitario 12 de Octubre
70. CEIC del Hospital Gregorio Marañón
71. Hospital Ramón y Cajal ;Comité Ético de investigación Clínica
72. Hospital Virgen del Rocío, CEIC
73. CEIC Hospital de Bellvitge
74. CEIC Corporació Sanitaria Parc Tauli
75. Comité Coordinador de Ética de la investigación Biomédica de Andalucía (CEIBA)
76. Hospital Clinic I Provincial; Comite Etico de Investigacion Clinica Chang Gung Med Found, Institutional Review Board
77. Research Ethics Committee, Nat. Taiwan Univ. Hosp.
78. The IRB, Taichung Veterans General Hospital
79. Siriraj Institutional Review Board
80. Ethical Clearance Committee on Human Rights
81. Songklanagarind Ethics Committee
82. Institutional Review Board, Faculty of Medicine
83. Research Ethics Com. Fae Med. Chiang May University
84. Trakya University Medical School Ethics Committee (for initial approval); Istanbul Uni. Cerrahpasa Medical School Ethics Committee (current EC due legislation change)
85. London Central Research Ethics Committee
86. Western Institutional Review Board
87. Cleveland Clinic Florida; Cleveland Clinic institutional Review Board
88. The University of Chicago IRB
89. Vanderbilt University Institutional Review Board
90. Memorial Sloan Kettering Cancer Center; Institutional Review Board
91. Dana Farber Cancer Institute Institutional Review Board
92. UC Irvine office of Research Administration
93. US Oncology, Inc Institutional Review Board
94. Copernicus Group IRB
